# Designing Imidazolium Poly(amide-amide) and Poly(amide-imide) Ionenes and Their Interactions with Mono- and Tris(imidazolium) Ionic Liquids

**DOI:** 10.3390/polym12061254

**Published:** 2020-05-30

**Authors:** Kathryn E. O’Harra, Danielle M. Noll, Irshad Kammakakam, Emily M. DeVriese, Gala Solis, Enrique M. Jackson, Jason E. Bara

**Affiliations:** 1 Department of Chemical & Biological Engineering, University of Alabama, Tuscaloosa, AL 35487-0203, USA; keoharra@crimson.ua.edu (K.E.O.); dmnoll@crimson.ua.edu (D.M.N.); ikammakakam@eng.ua.edu (I.K.); emdevriese@crimson.ua.edu (E.M.D.); 2 NASA Marshall Space Flight Center, Huntsville, AL 35812, USA; gala.c.solis@nasa.gov (G.S.); enrique.m.jackson@nasa.gov (E.M.J.)

**Keywords:** ionenes, copolymers, polyamides, polyimides, polyelectrolytes, composites

## Abstract

Here we introduce the synthesis and thermal properties of a series of sophisticated imidazolium ionenes with alternating amide-amide or amide-imide backbone functionality, and investigate the structural effects of mono(imidazolium) and unprecedented tris(imidazolium) ionic liquids (ILs) in these ionenes. The new set of poly(amide-amide) (PAA) and poly(amide-imide) (PAI) ionenes represent the intersection of conventional high-performance polymers with the ionene archetype–presenting polymers with alternating functional and ionic elements precisely sequenced along the backbone. The effects of polymer composition on the thermal properties and morphology were analyzed. Five distinct polymer backbones were synthesized and combined with a stoichiometric equivalent of the IL 1-benzyl-3-methylimidazolium bistriflimide ([Bnmim][Tf_2_N]), which were studied to probe the self-assembly, structuring, and contributions of intermolecular forces when IL is added. Furthermore, three polyamide (PA) or polyimide (PI) ionenes with simpler xylyl linkages were interfaced with [Bnmim][Tf_2_N] as well as a novel amide-linked tris(imidazolium) IL, to demonstrate the structural changes imparted by the inclusion of functional, ionic additives dispersed within the ionene matrix. This work highlights the possibilities for utilizing concepts from small molecules which exhibit supramolecular self-assembly to guide creative design and manipulate the structuring of ionenes.

## 1. Introduction

**Figure 1:**
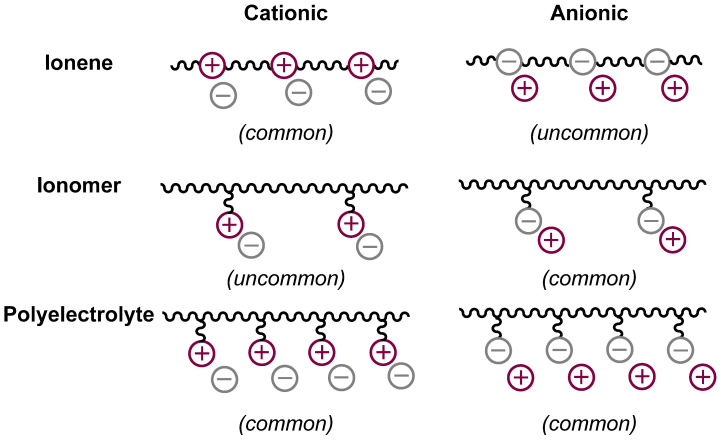
General representation of ionic polymer architectures, indicating their prevalence in literature.

Ionic polymers present versatile structural and functional possibilities, vast options for ionic or ionizable groups and respective counterion types, and utility in diverse applications. The features and behaviors of ionic polymers vary significantly, depending on the spacing and location (i.e., pendant or within chain) as well as the nature (i.e., anionic, cationic, zwitterionic) of charged moieties, which also impact the intermolecular interactions and characteristics contributed by other functional and structural features. The classification of polyelectrolytes, ionomers, and ionenes distinguish the concentration and location of charged groups and further support innumerable structural possibilities within ionic polymer design (Figure 1). There have been abundant research efforts in recent decades dedicated to the design and applications surrounding polyelectrolytes and ionomers which contain charged features pendant to the main chain, highlighted in several reviews [1,2,3,4,5,6]. The design of ionic liquids (ILs) undoubtably serves as an influential synthetic inspiration for polyelectrolytes, which possess essentially limitless tunability structurally through manipulation of features including the chemical structure of the ionic group, paired counterion, charge density, and proximity to the possibly functional main chain. However, ionenes, polymers which contain ionic groups within the backbone, are a class of materials that can be constructed utilizing established synthetic approaches and are compatible with many functional and structural elements associated with classical condensation polymers. Our recent review highlights the progress of ionenes and their convergence with high-performance applications [7].

The utility of ionenes has been demonstrated in separations and CO_2_ capture [8,9,10,11,12,13], ion-exchange or conducting membranes [14], antimicrobial coatings [15,16,17,18], batteries [19,20,21], electrochromic devices [22], hydrogels and gelators [23,24,25], water treatment [26], and fiber applications [12,27,28]. Nearly all known ionenes bear cationic moieties such as ammonium, phosphonium, pyrrolidinium, pyridinium, triazolium, and imidazolium groups [8,14,20,22,23,29,30,31,32,33,34]. While variation of cationic and anionic groups in polyelectrolytes have been studied, there is limited work probing the specific effects of cation and anion variation in ionenes [35,36,37]. Long’s group has emphasized the progress and versatile applicability of ammonium, imidazolium, and phosphonium ionenes [32,38,39]. Previous literature by Bara and coworkers has demonstrated the expansive possibilities of imidazolium ionenes, shifting toward more “high-performance” materials and exploring the effects of introducing imidazolium ILs into the ionene matrix [9,10,13,28,36,40,41]. We have demonstrated the suitability of imidazolium ionenes applied as CO_2_-selective gas separation membranes, self-healing and shape memory materials, 3D printing feedstocks, and as fibers [9,10,11,12,13,27,28]. The tailorability of this imidazolium platform is vast, when creative chemistries are employed to probe the effects of structural or connective variation of the cyclic cation, substitution, charge density, functionality, and modification of properties via addition of ILs. 

Despite the expansive design opportunities and modularity of synthetic routes, limited linkages, structural variations, functional features bridging ionic units have been exploited within ionenes and composites. Most ionenes reported primarily consist of ionic groups tethered with hydrocarbon or PEG-type linkages, though some derivatives have been reported with more aromatic character or robust functionality which impart greater thermal and mechanical stability [7]. The counterion affects thermal stability, rheological properties, and other behaviors in cationic ionenes, and several anions from simple halides to larger anions including PF_6_^−^, BF_4_^−^, or Tf_2_N^−^ have been paired within imidazolium ionenes [25,35,36,37]. While some “segmented” or random copolymer-like ionenes have been reported, expansion of synthetic approaches to achieve alternating yet distinct functionality among charged features in ionenes has been minimally explored [31,33,42,43,44,45].

Self-assembly and the structuring of molecules has undoubtably been of interest in ILs and has been previously studied [46,47,48,49,50]. Yet, the experimentation with the addition of monovalent ILs to ionenes and the effects on structure and properties has only recently emerged. This concept has not been explored beyond simple ILs, or established with multivalent species. Drawing inspiration from supramolecular chemistry, small molecules which possess intermolecular interactions as a result of ionic or H-bonding interactions self-assemble and these concepts could contribute toward ordering the ionene matrix. Benzene-1,3,5-carboxamides (BTA) are an intriguing moiety for building more complex ILs to include in ionenes, as BTA exhibits defined, 1-dimensional helical assembly as a result of stacking and stabilization via three-fold H-bonding [51,52]. It was shown that chirality could be imposed based on the side chains and altered by structural features at the periphery. The evolution of tunable, supramolecular simple molecules can be utilized as guidance toward structural design of ordered polymeric materials and the development of self-assembled composites. 

Only a few examples of polyamide (PA) ionenes have been demonstrated in literature, typically containing ammonium, pyridinium, and imidazolium cations. Our group has introduced aliphatic and aromatic imidazolium-based PA ionenes used as gas separation membranes or self-healing elastomers [10,12,27,28]. We have also recently demonstrated that some of the key monomers for these PA ionenes can be derived from poly(ethylene terephthalate) (PET). Other PA ionenes have been highlighted for their antimicrobial properties and as gelators for both water (hydrogels) or ILs (ionogels) [24,25,53]. Yoshido and Misawa investigated the gelation behavior of PA ionenes, where a diamide-dichloride linkage was utilized to form ammonium ionenes [24,53]. Waser and coworkers synthesized ammonium ionenes utilizing this diamide dichloride link, with resultant materials used to catalyze CO_2_-fixation with epoxides [54]. Yet, the use of this amide-functionalized dihalide in ionenes remains limited to small, simple co-monomers, including DABCO or N,N,N’,N’-tetramethyl-diamino-alkanes to form gels, some of which also exhibited self-healing behavior [25,55,56,57]. 

Herein we demonstrate the expansion of functional, robust ionenes to include mixed poly(amide-amides) (PAA) and poly(amide-imides) (PAI) utilizing this dichloride diamide linkage with a series of bis-imidazoles. While mixed functionality has been shown in ionenes, this typically is achieved by a stoichiometric balance of different monomers with the same end groups (random copolymerization), or the preparation of pre-polymers or oligomers which are linked secondarily. This series serves as an original example of designing tailored A-A and B-B building blocks for accessing more sophisticated ionenes, with distinct functionality and comparable structural complexity in each monomer. This approach enables the formation homogenous polymers with precisely alternating functional groups, because of the incorporation of amide or imide groups within bis-imidazole and dihalide monomers which are subsequently polymerized in one step to quaternize imidazolium groups via the Menshutkin reaction. These methods allow for the controlled sequencing and spacing of functionality using only two monomers, with distributed ionic groups located between these features. In our previous work, we have introduced multiple series of PA and PI ionenes, and reported that these ionene systems undergo ordering when “free” imidazolium IL is present [9,10,11,12,27,28,36]. This free IL serves as a non-covalent crosslink between ionene chains. The effects of IL on these PAA and PAI was studied through the addition of stoichiometric equivalents of 1-benzyl-3-methylimidazolium bistriflimide ([Bnmim][Tf_2_N]). Our previous work demonstrated that [Bnmim][Tf_2_N] imparted greater structural changes to the ionene matrix, in comparison with [C_n_mim][Tf_2_N] ILs, plausibly because of the combined effects of the imidazolium and aromatic features affecting the intermolecular forces between ionene chains [9,10]. Investigation of these IL composites was expanded through the design of a novel, BTA trisimidazolium IL (BTA(MeIm^+^)_3_][Tf_2_N]_3_, which was added to xylyl-linked PA and PI ionenes. This unprecedented work signifies the possibilities of adding functionality via trivalent IL fillers which directly alter intermolecular interactions, interchain spacing, and processability. The structural and functional modifications yielded robust, high-molecular weight polymers which demonstrate promise in various engineering applications. These studies emphasize the modularity of ionene design, controlled by analysis and understanding of which “knobs to turn” in order to tailor the structure and behaviors of sophisticated ionenes. 

## 2. Materials and Methods

### 2.1. Materials

Terephthaloyl chloride (TC, >99%), 1-(3-aminopropyl)imidazole) (API, >97), benzene-1,3,5-tricarbonyl trichloride (>98%) and 4-(chloromethyl)benzoyl chloride (>98%) were purchased from TCI (Tokyo, Japan). 1,4-phenylenediamine (>97%) was purchased from Alfa Aesar (Haverhill, MA, USA). Potassium carbonate (K_2_CO_3,_ 99% anhydrous) and iodomethane (99.5% stabilized) were purchased from BeanTown Chemical (Hudson, NH, USA). Lithium bistriflimide (LiTf_2_N, 99%) was purchased from 3M (Minneapolis, MN, USA). N-methylpyrrolidone (NMP, ACS grade), N,N-dimethylacetamide (DMAc, ACS grade), N,N-dimethylformamide (DMF, anhydrous), toluene (anhydrous), dichloromethane (DCM, anhydrous), triethylamine (Et_3_N), and acetonitrile (CH_3_CN, ACS grade) were purchased from VWR (Atlanta, GA, USA). 4,4′-(hexafluoroisopropylidene)diphthalic anhydride (6FDA, 99%) and pyromellitic dianhydride (PMDA, 99%) were purchased from Akron Polymer Systems (Akron, OH, USA). All reagents and solvents were utilized as obtained, without further purification.

### 2.2. Characterization

^1^H NMR data were obtained using 360 MHz or 500 MHz Bruker Avance instruments (Billerica, MA, USA). FTIR data was collected using a Perkin Elmer Spectrum Two ATR-FTIR instrument (Waltham, MA, USA). The thermal stabilities of these ionenes were evaluated by thermogravimetric analysis (TGA) at a heating rate of 10 °C.min^−1^ under a nitrogen atmosphere (Seiko TG/DTA7300, Chiba, Japan). The glass transition temperature (T_g_) of each ionene was observed by differential scanning calorimetry (DSC) (TA Instruments DSC Q20, New Castle, DE, USA) from 20 to 300 °C with a scan rate of 10 °C.min^−1^ under N_2_. The wide-angle X-ray diffraction patterns of the materials were measured using a Bruker D8 Discover diffractometer (Billerica, MA, USA) by employing a scanning rate of 4° min^−1^ in a 2*θ* range from 17° to 70° with a Co K*α*1 X-ray (*λ* = 0.17886 nm) source. The d-spacing values were calculated using Bragg’s law (d = *λ*/(2sin *θ*)). Number-average molecular weight (M_N_) values were determined via matrix assisted laser desorption/ionization time of flight mass spectrometry (MALDI-TOF MS) (Bruker Rapiflex, Billerica, MA, USA). Density data were collected for each ionene composite. The density measurements were performed using a Mettler Toledo Density Kit (Product MS-DNY-54, Columbus, OH, USA), paired with a Mettler Toledo analytical balance. This kit allows for solid state density determinations based on Archimedes’ principle. Bulk samples were prepared and dried thoroughly, then tested in triplicate. The samples were weighed at room temperature in air and then in a liquid of known density, in this case heptane. Note that these ionenes and ILs are completely insoluble and show no swelling/solvent interactions with heptane. The measurement was performed using the buoyancy method, and calculations were based on the following equation,

(1) $$\rho_{polymer}=\frac{W_{0}}{W_{0}-W_{1}}\times \rho_{liquid\;}$$

where *W*_0_ and *W*_1_ are the membrane weights in air and heptane, respectively. The uncertainty for these measurements is ± 0.01 g [10,58]. Magnified film images were obtained using a Leica DM2700 polarized light microscope (PLM) (Wetzler, Germany). 

### 2.3. Synthesis of Monomers and Small Molecules

#### 2.3.1. Synthesis of Bis(imidazoles)

**Scheme 1:**
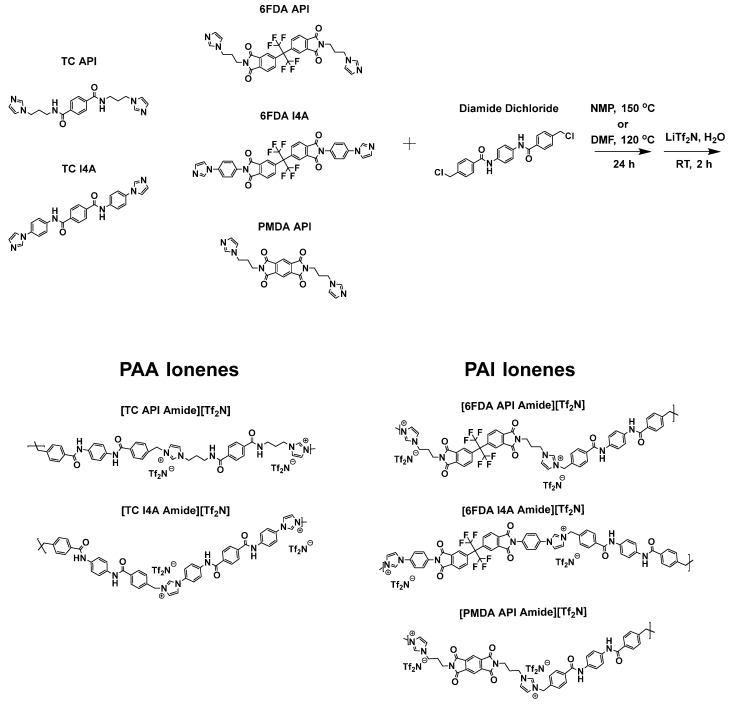
Synthesis of poly(imide-amide) and poly(amides) from bis(imidazole) and diamide dichloride linkage, polymerized via the Menshutkin reaction.

Synthetic procedures for all five imide or amide functionalized bis(imidazole) monomers were outlined in prior works [9,11,13,28,36]. The corresponding structures are shown in Scheme 1. “6FDA API” and “6FDA I4A” were formed via condensation of 6FDA with API or 4-(1H-imidazol-1-yl)aniline (“I4A”) in DMF. “PMDA API” was formed via condensation of PMDA with API in DMF. “TC API” and “TC I4A” were formed via condensation of API or I4A in the presence of K_2_CO_3_ in CH_3_CN. 

#### 2.3.2. Synthesis of N,N’-(1,4-phenylene)bis(4-(chloromethyl)benzamide) “Diamide Dichloride Linkage”

The diamide dichloride linkage, *N,N’*-(1,4-phenylene)bis(4-(chloromethyl)benzamide), was formed via the condensation of 4-(chloromethyl)benzoyl chloride and *p*-phenylenediamine in DCM with Et_3_N according to established literature procedures [24,53]. ^1^H NMR (500 MHz, DMSO-d_6_) δ 10.29 (s, 2H), 7.97 (d, J = 7.0 Hz, 4H), 7.76 (s, 4H), 7.60 (d, J = 7.27 Hz, 4H), 4.85 (s, 4H).

#### 2.3.3. Synthesis of N1,N3,N5-tris(4-(1H-imidazol-1-yl)phenyl)benzene-1,3,5-tricarboxamide and 1,1’,1’’-(((benzene-1,3,5-tricarbonyl)tris(azanediyl))tris(benzene-4,1-diyl))tris(3-methyl-1H-imidazol-3-ium) “[BTA(MeIm^+^)_3_][Tf_2_N^‒^]_3_”

**Scheme 2:**
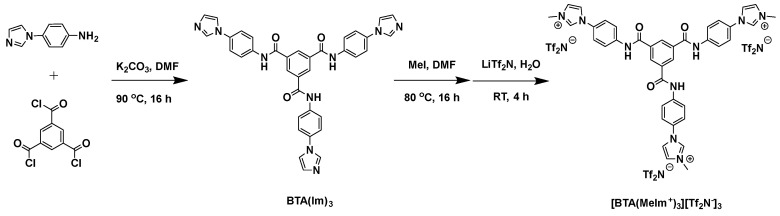
Synthesis of BTA(Im)_3_ and [BTA(MeIm^+^)_3_][Tf_2_N^‒^]_3_ filler.

The synthesis of the benzene-1,3,5-carboxamide trisimidazolium bistriflimide ([BTA(MeIm^+^)_3_][Tf_2_N^‒^]_3_) filler was a two-stage process, detailed in Scheme 2, involving a condensation reaction to form the amide functionality followed by alkylation to quaternize the peripheral imidazoles to the imidazolium form. 4-(1H-imidazol-1-yl)aniline, or “I4A”, has been introduced in several of our previous works [9,10,13]. Benzene-1,3,5-tricarbonyl trichloride (2.00 g, 7.53 mmol), I4A (3.78 g, 23.73 mmol), and K_2_CO_3_ (3.28 g, 23.73 mmol) were added with 50 mL DMF to a round-bottom heavy-walled 100 mL flask (Ace Glass, Vineland, NJ, USA). The reaction was sealed with a PTFE screw cap and heated to 90 °C for 16 h. The solution was poured into 250 mL of chilled DI H_2_O to precipitate the neutral product. The solids were filtered and washed with water, then dried in a vacuum oven for 16 h at 105 °C, to yield the “TA(Im)_3_” product as an off-white powder (3.74 g, 78%). ^1^H NMR (500 MHz, DMSO-d_6_) δ 10.75 (s, 3H), 8.69 (s, 3H), 8.25 (s, 3H), 7.99 (d, J = 7.39 Hz, 6H), 7.68 (m, J = 8.24, 11.33 Hz, 9H). 

The neutral form was used directly for the next step. BTA(Im)_3_ (1.00 g, 1.58 mmol), was added again to a 100 mL round-bottom heavy-walled flask with 40 mL DMF, and excess iodomethane (0.59 mL, 9.47 mmol) was carefully added via syringe. The reaction was sealed with a PTFE screw cap allowed to stir at room temperature (RT) for 1 h, then was heated to 80 °C for 16 h. The reaction was cooled to RT, and precipitated into a DI H_2_O containing LiTf_2_N (4 eq., 1.81 g) to promote anion metathesis from the I^‒^ to Tf_2_N^‒^ form. The ([BTA(MeIm^+^)_3_][Tf_2_N^‒^]_3_ product was filtered and washed with DI H_2_O (100 mL × 2) then diethyl ether (100 mL × 2). The product was isolated as a tan powder (1.846 g, 76%). ^1^H NMR (500 MHz, DMSO-d_6_) δ 10.95 (br, 3H), 9.73 (s, 3H), 8.80 (d, J = 10.95 Hz, 3H), 8.28 (m, J = 1.52 Hz, 3H), 8.08 (td, J = 1.21, 8.85 Hz, 6H), 7.96 (m, 3H), 7.81 (td, J = 1.20, 8.76, 6H) 3.97 (s, 9H).

### 2.4. Synthesis of Poly(amide), Poly(imide) and Poly(imide-amide) Ionenes

This series of 5 PAA and PAI ionenes were synthesized via the Menshutkin reaction between various imide or amide-functionalized bis-imidazole monomers and the aforementioned diamide dichloride. The series of xylyl-linked PA and PI ionenes interfaced with IL and the amide functionalized tris-imidazolium bistriflimide filler: [TC API pX][Tf_2_N], [TC I4A pX][Tf_2_N], and [PMDA API pX][Tf_2_N] were synthesized according to procedures reported in our previous work [10,11,12,28,36]. It should be highlighted that as the halide (i.e., Cl^−^) salt, these ionenes are all water soluble. However, each becomes hydrophobic upon exchange to the Tf_2_N^‒^ salt. 

#### 2.4.1. Synthesis of [TC API Amide][Tf_2_N]

TC API (3.00 g, 7.89 mmol) and the diamide dichloride (3.259 g, 7.89 mmol) were added with 30 mL NMP to a 100 mL heavy-walled round-bottom pressure vessel (Ace Glass, Vineland, NJ, USA) equipped with a stir bar. The reaction was stirred at 150 °C for 24 h. The reaction was cooled to RT and a portion of the ionene precipitated from solution as the Cl^−^ salt. The ionene was precipitated in DI H_2_O with 2.5 eq. LiTf_2_N (5.66 g) to promote anion metathesis from the Cl^−^ to the Tf_2_N^−^ salt. The product was filtered and dried in a vacuum oven at 105 °C overnight to yield a light brown gel (8.2 g, 81%). ^1^ H NMR (500 MHz, DMSO-d6) δ 10.35 (s, 2H), 9.47 (s, 2H), 8.87 (s, 2H), 8.04 (d, J = 7.19 Hz, 4H), 7.99 (s, 4H), 7.93 (s, 2H), 7.87 (s, 2H), 7.77 (s, 4H), 7.58 (d, J = 7.24 Hz, 4H), 5.55 (s, 4H). 4.30 (br, 4H), 3.37 (br, 4H), 2.13 (br, 4H). 

#### 2.4.2. Synthesis of [TC I4A Amide][Tf_2_N]

TC I4A (1.570 g, 3.5 mmol) and the diamide dichloride (1.45 g, 3.5 mmol) were added with 30 mL NMP to a 100 mL heavy-walled round-bottom pressure vessel (Ace Glass) equipped with a stir bar. The reaction was stirred at 150 °C for 24 h. The ionene solids precipitated from solution as the Cl^−^ salt during the reaction, and was subsequently cooled to RT. The ionene was precipitated in deionized (DI) H_2_O with 2.5 eq. LiTf_2_N (2.51 g) to promote anion metathesis from the Cl^−^ to the Tf_2_N^−^ salt. The product was filtered and dried in a vacuum oven at 105 °C overnight to yield a tan gel (3.4 g, 72%). ^1^H NMR (500 MHz, DMSO-d6) δ 10.78 (s, 2H), 10.33 (br, 2H), 10.08 (br, 2H), 8.38 (br, 2H), 8.19 (d, J = 3.94 Hz, 4H), 8.08 (q, J = 4.74, 9.11 Hz, 8H), 7.86 (d, J = 8.27 Hz, 4H), 7.78 (br, 6H), 7.69 (d, J = 8.68 Hz, 4H), 5.63 (br, 4H). 

#### 2.4.3. Synthesis of [6FDA API Amide][Tf_2_N]

6FDA API (2.00 g, 3.03 mmol) and the diamide dichloride (1.25 g, 3.03 mmol) were added with 30 mL DMF to a 100 mL heavy-walled round-bottom pressure vessel (Ace Glass, Vineland, NJ, USA) equipped with a stir bar. The reaction was stirred at 120 °C for 24 h. The ionene precipitated as a gel from solution as the Cl^−^ salt during the reaction, and was subsequently cooled to RT. The ionene was precipitated in DI H_2_O with 2.5 eq. LiTf_2_N (2.17 g) to promote anion metathesis from the Cl^−^ to the Tf_2_N^−^ salt. The product was filtered and dried in a vacuum oven at 105 °C overnight to yield a brown glassy solid (3.16 g, 67%). ^1^H NMR (500 MHz, DMSO-d6) δ 10.29 (s, 2H), 9.30 (s, 2H) 8.11 (d, J = 7.79 Hz, 2H), 8.01 (d, J = 7.02 Hz, 2H), 7.95 (m, 6H), 7.90 (br, 2H), 7.80 (s, 4H) 7.73 (s, 4H), 7.75 (s, 4H), 7.57 (d, J = 7.34 Hz, 4H), 5.55 (s, 4H). 4.29 (br, 4H), 3.65 (br, 4H), 2.20 (br, 4H).

#### 2.4.4. Synthesis of [6FDA I4A Amide][Tf_2_N]

6FDA I4A (1.00 g, 1.38 mmol) and the diamide dichloride (0.569 g, 1.38 mmol) were added with 30 mL DMF to a 100 mL heavy-walled round-bottom pressure vessel (Ace Glass, Vineland, NJ, USA) equipped with a stir bar. The reaction was stirred at 120 °C for 24 h. The ionene precipitated as a gel from solution as the Cl^−^ salt during the reaction, and was subsequently cooled to RT. The ionene was precipitated in DI H_2_O with 2.5 eq. LiTf_2_N (0.99 g) to promote anion metathesis from the Cl^−^ to the Tf_2_N^−^ salt. The product was filtered and dried in a vacuum oven at 105 °C overnight to yield a brown glass (1.48 g, 66%). ^1^H NMR (500 MHz, DMSO-d6) δ 10.35 (s, 2H), 10.11 (s, 2H), 8.43 (s, 2H), 8.26 (d, J = 6.98 Hz, 2H), 8.12 (s, 2H), 8.06 (d, J = 6.79 Hz, 4H), 8.01 (d, J = 6.90 Hz, 6H), 7.79 (br, 2H), 7.78 (br, 8H), 7.70 (d, J = 6.86 Hz, 4H), 5.65 (s, 4H). 

#### 2.4.5. Synthesis of [PMDA API Amide][Tf_2_N]

PMDA API (1.514 g, 3.5 mmol) and the diamide dichloride (1.45 g, 3.5 mmol) were added with 30 mL NMP to a 100 mL heavy-walled round-bottom pressure vessel (Ace Glass, Vineland, NJ, USA) equipped with a stir bar. The reaction was stirred at 150 °C for 24 h. The ionene solids precipitated from solution as the Cl^−^ salt during the reaction, and was subsequently cooled to RT. The ionene was precipitated in DI H_2_O with 2.5 eq. LiTf_2_N (2.51 g) to promote anion metathesis from the Cl^−^ to the Tf_2_N^−^ salt. The product was filtered and dried in a vacuum oven at 105 °C overnight to yield a dark brown solid (2.74 g, 59%). ^1^H NMR (500 MHz, DMSO-d6) δ 10.33 (br, 2H), 9.38 (br, 2H), 8.24 (br, 2H), 8.03 (d, J = 7.01 Hz, 4H), 7.86 (s, 4H), 7.76 (s, 4H), 7.57 (d, J = 7.41 Hz, 4H) 5.55 (br, 4H). 4.30 (br, 4H), 3.69 (br, 4H), 2.24 (br, 4H). 

### 2.5. Preparation of Ionene: [Bnmim][Tf_2_N] and Ionene: [BTA(MeIm^+^)_3_][Tf_2_N^‒^]_3_ Composites

**Scheme 3:**
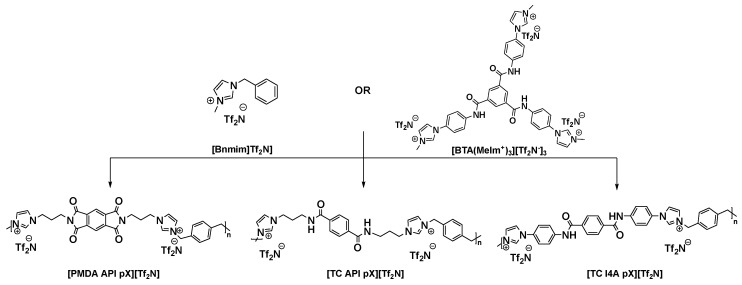
Structures of additives, [Bnmim][Tf_2_N] and [BTA(MeIm^+^)_3_][Tf_2_N^‒^]_3_, incorporated into neat polyimide (PI) and polyamide (PA) ionenes.

To ensure uniform dispersion of IL or filler within each ionene, composites were prepared in solution, cast, and then the solvent was removed to yield a homogenous composite. Stoichiometric equivalents of these additives were added to the respective ionenes shown in Scheme 3. For example, 2 equivalents of IL were added per repeat unit (RU) of the corresponding PAA or PAI ionenes, as each RU contains two imidazolium bistriflimide groups (Table 1). Since the [BTA(MeIm^+^)_3_][Tf_2_N^‒^]_3_ filler contains 3 imidazolium bistriflimide groups per molecule and each xylyl linked PA or PI ionene contains 2 in the RU, the stoichiometric ratio of ionene to [BTA(MeIm^+^)_3_][Tf_2_N^‒^]_3_ was 3:2. 

**Figure 2:**
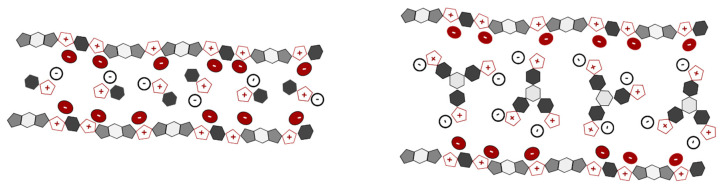
Graphic representing the incorporation of mono- and tris-imidazolium ionic liquid (IL) within the ionene matrix. The cartoon structures refer to the polymer and IL structures shown in Scheme 3. For example, pentagon are five-membered rings, hexagons are six-membered rings and circles are anions.

For the amide-linked PAA and PAI series, 0.5 g of ionene was added to a 15 mL centrifuge tube with 2 eq. of [Bnmim][Tf_2_N]. DMAc (8 mL) was added to each tube, and all five solutions were periodically agitated and heated in a water bath over 48 h to ensure dissolution of the ionene. While still warm, the tubes were centrifuged at 6000 rpm for 3 min to ensure settling of any undissolved particulates. The solutions were then cast into shallow 60 mm PTFE wells, and dried in a vacuum oven at 120 °C for 48 h to evaporate the solvent. The xylyl-linked PA and PI composites were prepared via a similar procedure, using 0.5 g ionene and the corresponding equivalents of [Bnmim][Tf_2_N] or [BTA(MeIm^+^)_3_][Tf_2_N^‒^]_3_ in 8 mL of acetone. Following dissolution and centrifugation, the composites were cast and dried for 24 h at 75 °C. Mass ratios for these composites are included in Table 2. A distinct feature of these composites is their opaque and paper-like texture which is in contrast to the typically transparent appearance of neat ionenes. Representation of the interactions between these ionenes and mono- and tris-imidazolium ILs is shown in Figure 2.

## 3. Results and Discussion

As mentioned, the formation of each amide-linked ionene and the BTA(Im)_3_/[BTA(MeIm^+^)_3_][Tf_2_N^−^]_3_ additives was confirmed using ^1^H-NMR. Expanded analysis, peak assignments, and spectra are included in Figures S1–S8 (Supplementary Materials). FTIR was also utilized to confirm the backbone functionality and associated anion in the five PAA and PAI ionenes (Figure S9, Supplementary Materials). The molecular weight, structural features, and thermal properties for this series of ionenes and composites were analyzed, in order to characterize the properties of these new ionenes and the effects of additives on structuring of the ionene matrix. 

### 3.1. Molecular Weight Confirmation

MALDI-TOF MS was used to determine the number average molecular weight (M_N_) and degree of polymerization (X_N_) for the five new PAA and PAI ionenes. MALDI-TOF MS has been utilized with our previous imidazolium ionenes [9,10,12,13,36] and by other groups characterizing cationic ionenes, in order to analyze molecular weight [59,60,61]. A concentrated sample was prepared with DMAc, and multiple μL-sized spots were deposited onto the plate with different matrices (THAP, DCTB in MeOH) added following drying. This confirmed that high molecular weight polymers were formed, showing M_N_ values in the range of 46.7–86.1 kDa with corresponding X_N_ values in the range of 34–67 repeat units. While this information is summarized in Table 1 and reported in Figure S10 (Supplementary Materials), it should be noted that [TC API pX][Tf_2_N] achieved the highest molecular weight. This is attributed to the fact that this derivative was the only ionene in the set which remained soluble throughout the reaction and thus was able to continue chain growth. These X_N_ values are consistent with those calculated end group analysis. The peak integration ratio of residual benzylic CH_2_Cl protons from the diamide dichloride linkage relative to the polymerized form is evident from the resultant shift in the ^1^H NMR, from ~4.85 ppm to ~5.4–5.65 ppm. These shifts are pronounced and clear, although integration and comparison of the shifting proton peaks for the imidazole to imidazolium transformation could also be analyzed. From these ratios, conversion (*ρ*) and thus X_N_ were derived using Carothers’ equation, with calculated X_N_ values ~33–67 repeat units (*ρ* ≈ 0.97–0.985).

### 3.2. Thermal Characterization

#### 3.2.1. Thermal Behavior of Mixed PAA and PAI Ionenes

The thermal transitions and degradation of the PAA and PAI ionenes was investigated using DSC and TGA, and the transitions of the PA and PI composites with [BTA(MeIm^+^)_3_][Tf_2_N^−^]_3_ were probed using DSC for comparison with the corresponding neat derivatives. 

Table 1 summarizes the glass transition temperatures (T_g_) of the amide-linked series, which ranged from 105–177 °C. These T_g_ endotherms were broad, but supported by the corresponding exotherms, which are clarified in Figures S11 and S12 (Supplementary Materials). Each ionene shows a broad melting transition, between 175–245 °C (Figure S12, Supplementary Materials). The T_g_ trends were reasonable, consistently showing higher glass transitions for aromatic versus aliphatic analogs, which was more pronounced for the PAA ionenes. [PMDA API Amide][Tf_2_N] and [6FDA API Amide][Tf_2_N] demonstrated comparable T_g_’s, with the planar PMDA derivative showing a slightly higher transition than the bent 6FDA derivative. In comparison with xylyl linked versions of these ionenes, the H-bonding and aromatic character contributed by the more complex diamide dichloride generally increased the T_g_ [10,12,36].

These ionenes exhibited excellent thermal stability using TGA, as shown in Figure S13 (Supplementary Materials). Mass loss at ~210 °C is attributed to trapped residual solvent. All derivatives demonstrated little mass loss below 300 °C, with T_d,onset_ in the range of 305–418 °C. The [TC I4A Amide][Tf_2_N] ionene possessed both the highest T_g_ and T_d,onset_, indicating the improved thermal resilience in the derivative containing the most aromatic character and H-bonding sites. The T_d,onset_ values were consistent with expected trends, as aromatic derivatives showed higher thermal stability than the corresponding aliphatic derivatives.

#### 3.2.2. Thermal Behavior of Xylyl PA and PI Ionenes with [BTA(MeIm^+^)_3_][Tf_2_N^−^]_3_

DSC was utilized to study the effects of adding the [BTA(MeIm^+^)_3_][Tf_2_N^−^]_3_ filler, which at 1519.18 g/mol is a substantial portion of the ionene matrix. Table 2 summarizes the thermal behaviors, with plots included as Figures S14 and S15 (Supplementary Materials). The [BTA(MeIm^+^)_3_][Tf_2_N^−^]_3_ additive shows an apparent melting endotherm at 66.9 °C, followed by a broad secondary endotherm at 94 °C. Of course, the addition of a small molecule filler depressed the T_g_ for each ionene, in comparison to neat analogs. Broad endotherms are observed, and a faint crystallization exotherm can be seen in the 50–70 °C range which may indicate crystallization of the filler within the matrix. The molecular weight of the [BTA(MeIm^+^)_3_][Tf_2_N^−^]_3_ additive is higher than that of each ionene RU, and thus significant impact on the thermal and rheological behavior and organization of domains is possible.

### 3.3. Structural Characterization

**Figure 3:**
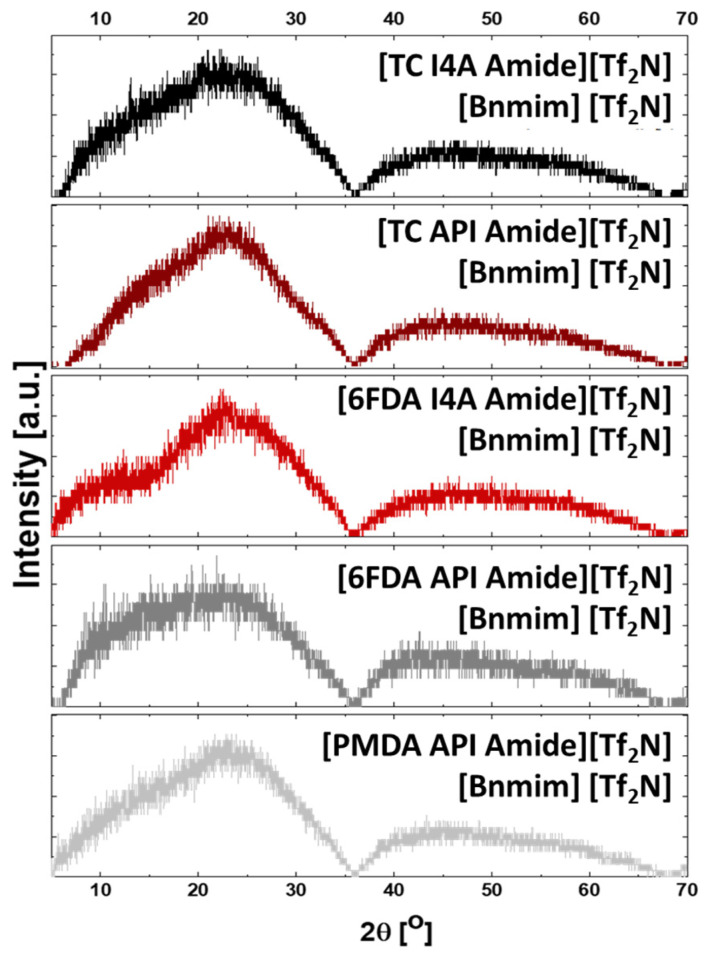
XRD profiles for all five PAA and PAI ionenes impregnated with 2 equivalents (eq.) of [Bnmim][Tf_2_N].

X-ray diffraction profiles for composite materials were analyzed, in order to study the effects of additives added to structure the ionene matrix. The intermolecular interactions within the ionene matrix are altered and pronounced by the addition of ionic fillers, which coordinate among charged chains. The d-spacing obtained from XRD has been correlated as an approximation of mean interchain spacing in amorphous polymeric materials, and the reported values are consistent with those of other polyamides and polyimides [26,62,63,64]. Our previous imidazolium-ionenes have shown comparable d-spacings, and typically display a sharpening or narrowing of the amorphous main “halo” with the addition of IL. The shape and maxima of the amide-linked series, shown in Figure 3 between 2θ = 5–36°, varies across each system. Because of the order imparted by stacking interactions, the more aromatic analogs show slightly narrower distributions than their aliphatic counterparts. The maximum 2θ was determined using Diffrac.Eva software, resulting in d-spacings in the range of 4.49–5.19 Å. 

**Figure 4:**
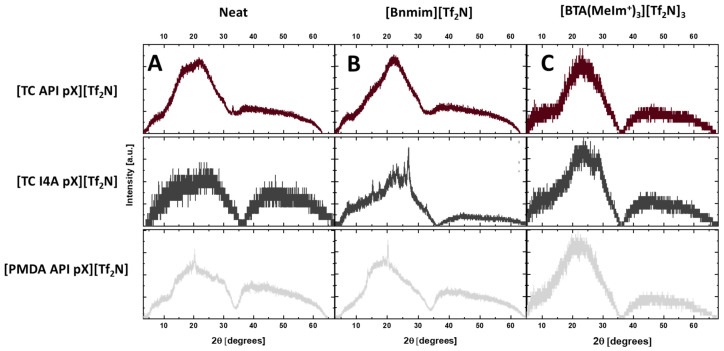
XRD profiles comparing all xylyl ionenes (**A**) neat, (**B**) with 2 equivalents of [Bnmim][Tf_2_N], and (**C**) containing stoichiometric equivalents of [BTA(MeIm^+^)_3_][Tf_2_N^−^]_3_.

Figure 4 shows the XRD profiles for the xylyl-linked series of ionenes neat, and with [Bnmim][Tf_2_N] or [BTA(MeIm^+^)_3_][Tf_2_N^−^]_3_. The shape of these profiles is clearly affected by both the mono- and tris-imidazolium bistriflimide composites in comparison to the corresponding neat ionenes, with a more defined progression toward a narrowed primary peak in the main halo. This insinuates that since the distribution of interchain spacings is less broad, the addition of IL or more complex fillers regulate the spacing between ionene chains. The main halo for all neat derivatives is broad, spanning 2θ ≈ 10–25°. It can be seen that the stoichiometric addition of [Bnmim][Tf_2_N] sharpens this peak, with resultant d-spacings between 3.64–6.71 Å, inducing more defined crystalline peaks when interfaced with the more aromatic [TC I4A pX][Tf_2_N] ionene. Finally, the [BTA(MeIm^+^)_3_][Tf_2_N^−^]_3_ filler resulted in clarification of the main peak. Additionally, a plateau between 2θ = 10–15° is observed in all derivatives containing [BTA(MeIm^+^)_3_][Tf_2_N^−^]_3_. The d-spacing values fall between 3.60–4.58 Å for these composites, as reported in Table 2. The [TC I4A pX][Tf_2_N] halo is bimodal, and thus the d-spacings for each maximum are given. These approximations and shifts could be utilized to predict the relative density and chain packing behavior, aid in understanding the design and coordination of dispersed additives, or potentially predict suitability for application as permeable but selective gas-separation membranes. The nature and proximity of the “free” IL or the concentration other ionic fillers may regulate interchain spacing or affect other properties such as ion conductivity. 

The density of the PAA and PAI composites with [Bnmim][Tf_2_N] as well as the PA and PI composites neat or with [BTA(MeIm^+^)_3_][Tf_2_N^−^]_3_, are reported in Table 1 and Table 2. For the PAA and PAI ionenes, the aliphatic ionenes were more dense than their corresponding aromatic derivatives, as the flexibility of the alkyl segments likely allow for closer chain packing and more chain entanglement than the bulkier, rigid segments in the aromatic analogs. Additionally, the PAAs were slightly less dense than the PAIs. The PA and PI ionenes were compared, neat or with [BTA(MeIm^+^)_3_][Tf_2_N^−^]_3_. The composite with [TC API pX][Tf_2_N] was more dense in comparison with the neat ionene, plausibly due to the strong H-bonding and ionic interactions among the flexible, elastomeric, and more aliphatic chains in this PA ionene. For the more rigid PA and PI, a decrease in density was observed with the addition of [BTA(MeIm^+^)_3_][Tf_2_N^−^]_3_, which suggests that the additive disrupts chain packing and entanglement in the less flexible ionenes.

### 3.4. Material Behavior and Potential Applicability

**Figure 5:**
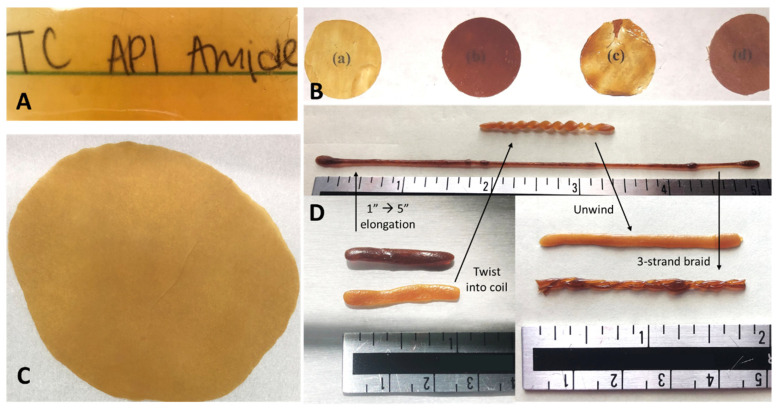
Images of PAA and PAI ionenes. (**A**) neat [TC API Amide][Tf_2_N] forms a transparent film from solvent casting; (**B**) other PAA and PAI ionenes {a: TC I4A Amide, b: PMDA API Amide, c: 6FDA API Amide, d: 6FDA I4A Amide} showing transparency as thin films; (**C**) [TC API Amide][Tf_2_N] as an opaque film melt-pressed at 120 °C; (**D**) [TC I4A Amide][Tf_2_N] and [6FDA API Amide][Tf_2_N] composite fibers, elongated to show elasticity and amenability to contortion (demonstrated through coiling or braiding).

The design and properties of these functional ionenes and composites illustrate promise in high-performance engineering applications, indicated by similar stabilities and mechanical character to prior PA and PI ionenes. Each derivative was amenable to formation of fibers or thin films (Figure 5). The defined thermal properties allow for processing techniques including melt-pressing, injection molding, or extrusion into fibers, which could be strengthened by alignment of alternating ionic and H-bonding participants specifically in the mixed PAA derivatives. While the neat PAA and PAI materials are relatively glassy and transparent, as shown in Figure 5A–D, the addition of IL results in apparent plasticization, which improved the flexibility and durability of the thin composite films. This shows potential for implementation as coatings or separation membranes, in addition to the good solubility in many organic solvents (DMF, NMP, DMAc, Acetone). This study elucidated the robust yet processible nature of the aromatic analogs, which may be most suitable for holding under pressure and thermal stresses in membrane-based separation processes. The aliphatic PAI derivatives and both PAAs are dense and tough, exhibiting greater flexibility and elastic character, which may indicate suitability as fibers (Figure 5D). Other properties such as conductivity or tensile and compressive response could be explored in the future.

**Figure 6:**
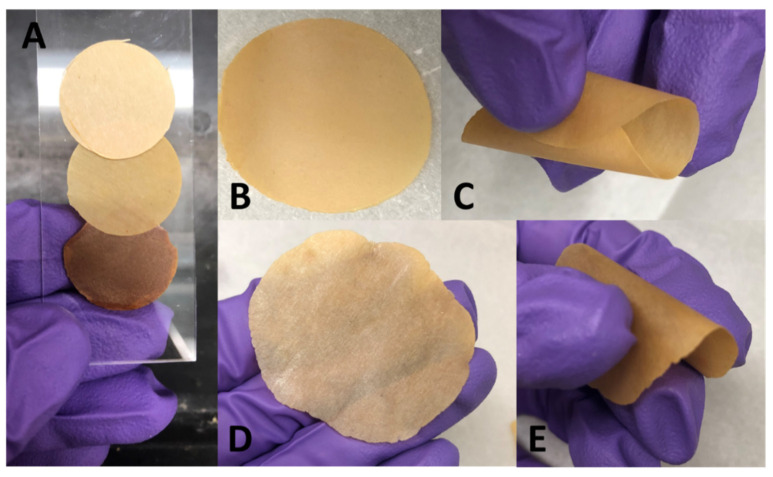
Images of ionenes containing [BTA(MeIm^+^)_3_][Tf_2_N^−^]_3_. (**A**) ¾” disks of composite films showing opacity; (**B**,**C**) [TC API pX][Tf_2_N]:[BTA(MeIm^+^)_3_][Tf_2_N^−^]_3_ cast as 2.5” disk, showing flexibility without creasing or adhesion, (**D**,**E**)) [TC I4A pX][Tf_2_N]:[BTA(MeIm^+^)_3_][Tf_2_N^−^]_3_ cast as 2.5” disk, showing flexibility when bent.

The introduction of a multivalent IL with amide groups complementary to those on the polymer designed to incorporate both ionic and functional content was shown to distribute well within the ionene matrix, indicating the amenability toward larger additives (than conventional ILs) with similar content to the polymeric framework. This demonstrates the possibilities of tuning interchain spacing and structuring domains within amorphous polymers using organic additives tailored to the material, which may aid in improving the permeability and selectivity of separation membranes without sacrificing homogeneity or stability. Figure 6A–E show that the [BTA(MeIm^+^)_3_][Tf_2_N^−^]_3_ filler imparted ordering within the ionic framework, inducing a leathery, opaque, and paper-like quality in previously transparent and amorphous neat ionenes, without affecting the flexibility of the freestanding, composite films. These composites are very flexible yet tough, which is promising for their stability under stresses such as pressure or contortion (Figure 6C,E).

**Figure 7:**
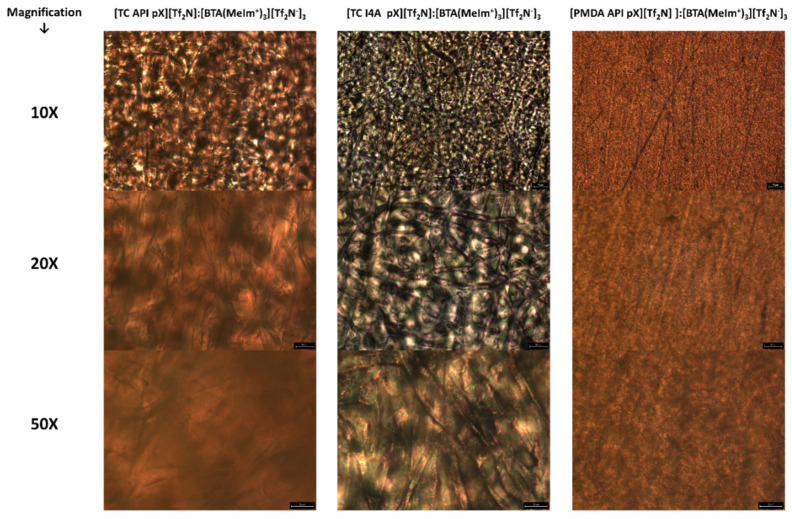
PLM images of PA and PI ionene composites with [BTA(MeIm^+^)_3_][Tf_2_N^−^]_3._ Materials showed ordered structures and were probed as thin films, at 10×, 20×, and 50× magnifications (scale bar, top to bottom: 75 μm, 50 μm, 25 μm).

The larger scale organization of the composite films shown in Figure 5 and Figure 6 was studied using a polarized light microscope (PLM). More rigid PAI composites showed more structural regularity than the PAAs containing IL (Figure S16, Supplementary Materials). More interesting behavior and ordering was observed in the [BTA(MeIm^+^)_3_][Tf_2_N^−^]_3_ composites when probed using the PLM. Figure 7 highlights the structuring imparted by this tris-imidazolium filler in each PA and PI derivative, showing the opaque thin films at magnifications of 10×, 20×, and 50×. The clarity of patterns and organization exhibited in these magnified images under polarized light may indicate stronger interactions and resultant assembly between the PA derivatives and the amide-functionalized ionic additive.

## 4. Conclusions

We have successfully synthesized new PAA and PAI imidazolium ionenes, with alternating amide and imide functionality, demonstrating the amenability and modularity of this route to produce sophisticated, copolymer-like ionenes with precisely spaced features. These amide-linked ionenes demonstrate the possibility of large, complex repeat unit design using only A-A + B-B monomers while retaining processability. The ionenes in this set are water soluble as Cl^−^ salts, yet robust and hydrophobic upon anion metathesis to the Tf_2_N form. Thermal characterizations supported the thermal stability of these PAA and PAI ionenes, and the structural effects of adding stoichiometric equivalents of imidazolium IL. Additionally, a set of xylyl-linked ionenes from our previous works were modified with the addition of IL as well as a novel, bulky filler containing “matching” functionality and ionic groups, which induce structuring within the ionene matrix. These concepts and materials demonstrate good processability from solvents or thermal stimuli, and wide potential applicability as thin films or fibers, because of the tunability of intermolecular interactions imparted by the combination of ionic, H-bonding, and aromatic versus aliphatic features. We seek to expand upon these design concepts and synthetic possibilities in future work, investigating mechanical behaviors and testing these materials in suitable applications.

## Figures and Tables

**Table 1:** Summary of characterization data for all five poly(amide-amide ) (PAA) and poly(amide–imide) (PAI) ionene derivatives.

Ionene	T_g_ (°C)	T_d, onset_ (°C)	$\bar{X_{N}}\;(\mathrm{RU})$	MW_RU_ (g/mol)	$\bar{M_{N}}\;(\mathrm{kDa})$	*d*-Spacing (Å)	Density (g/cm^3^)
	Neat Ionene					Ionene:IL Composite	
[6FDA API Amide][Tf_2_N]	139	363	35	1561.23	53.8	4.49	1.57
[6FDA I4A Amide][Tf_2_N]	160	392	34	1629.26	55.7	5.19	1.55
[TC API Amide][Tf_2_N]	105	333	67	1283.12	86.1	4.52	1.49
[TC I4A Amide][Tf_2_N]	177	418	44	1351.16	60.1	4.52	1.40
[PMDA API Amide][Tf_2_N]	145	305	35	1335.11	46.7	4.56	1.52

**Table 2:** Summary of characterization data for PA and PI composites with [Bnmim][Tf_2_N] and [BTA(MeIm^+^)_3_][Tf_2_N^−^]_3_, and composite compositions.

Ionene: IL	T_g_ (°C)	T_m_ (°C)	*d*-Spacings (Å)	Density (g/cm^3^)	MW_RU_ (g/mol)	MW_Add_ (g/mol)	Ionene:IL Mass (g)
[TC API pX][Tf_2_N]:Neat	82	130	4.70	1.49	1044.88	-	-
[TC I4A pX][Tf_2_N]:Neat	136	-	4.26	1.52	1112.91	-	-
[PMDA API pX][Tf_2_N]:Neat	91	190	5.08	1.66	1096.86	-	-
[TC API pX][Tf_2_N]:[Bnmim][Tf_2_N]	-	-	4.69	-	1044.88	453.37	0.5:0.43
[TC I4A pX][Tf_2_N]:[Bnmim][Tf_2_N]	-	-	3.8/4.5/5.9/6.7	-	1112.91	453.37	0.5:0.41
[PMDA API pX][Tf_2_N]:[Bnmim][Tf_2_N]	70	144	5.10/5.56	-	1096.86	453.37	0.5:0.41
[TC API pX][Tf_2_N]:[BTA(MeIm^+^)_3_][Tf_2_N^−^]_3_	38	160	4.45	1.59	1044.88	1519.18	0.5:0.48
[TC I4A pX][Tf_2_N]:[BTA(MeIm^+^)_3_][Tf_2_N^−^]_3_	52	-	3.60/4.49	1.49	1112.91	1519.18	0.5:0.46
[PMDA API pX][Tf_2_N]:[BTA(MeIm^+^)_3_][Tf_2_N^−^]_3_	70	155	4.58	1.56	1096.86	1519.18	0.5:0.46
Neat [BTA(MeIm^+^)_3_][Tf_2_N^−^]_3_	-	66.9	-	-	-	1519.18	-

## Supplementary Materials

The following are available online at https://www.mdpi.com/2073-4360/12/6/1254/s1, Figures S1–S5: PAA and PAI ionene ^1^H-NMR spectra, Figure S6: Diamide dichloride ^1^H-NMR spectrum, Figures S7 and S8: ^1^H-NMR spectra for BTA(Im)_3_ and [BTA(MeIm^+^)_3_][Tf_2_N^‒^]_3_, Figure S9: FTIR data for PAA and PAI ionenes, Figure S10: MALDI-TOF MS for PAA and PAI ionenes, Figures S11 and S12: DSC data for PAA and PAI ionenes, Figure S13: TGA data for PAA and PAI ionenes, Figures S14 and S15: DSC Data for PA and PI composites with [BTA(MeIm^+^)_3_][Tf_2_N^‒^]_3_, Figure S16: PLM images for PAA and PAI composites with [Bnmim][Tf_2_N].
