# Supplementary material for: Designing Imidazolium Poly(amide-amide) and Poly(amide-imide) Ionenes and Their Interactions with Mono- and Tris(imidazolium) Ionic Liquids
Source: Polymers (Basel). 2020 May 30;12(6):1254. doi: 10.3390/polym12061254 (PMC7362236; doi:10.3390/polym12061254)
Supplement: Supplementary file 1 [file polymers-12-01254-s001.pdf]

polymers

MDPI

Supplementary Materials

# Designing Imidazolium Poly(amide-amide) and Poly(amide-imide) Ionenes and Their Interactions with Mono- and Tris(imidazolium) Ionic Liquids

## 1. H-NMR Data:

The NMR data for all five monomers has been published in our previous work. The proton NMR spectra for the five ionenes and diamide dichloride linkage are included below, with the corresponding integrations and ppm details reported in the experimental section of the manuscript. Each spectrum includes the respective structure, labeled with corresponding peak assignments.

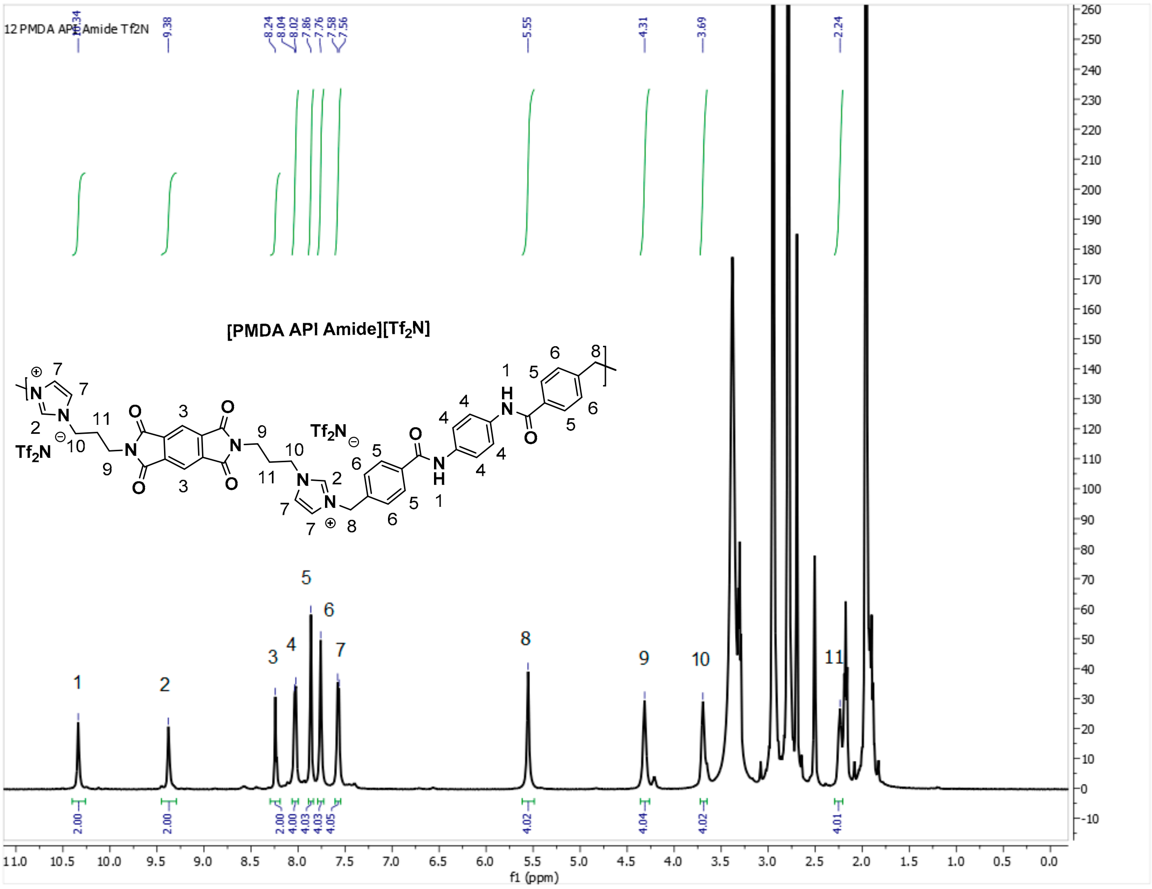


**Figure S1.** ¹H-NMR spectra for [PMDA API Amide][Tf₂N].

¹H NMR (500 MHz, DMSO-d6) δ 10.33 (br, 2H), 9.38 (br, 2H), 8.24 (br, 2H), 8.03 (d, J = 7.01 Hz, 4H), 7.86 (s, 4H), 7.76 (s, 4H), 7.57 (d, J = 7.41 Hz, 4H) 5.55 (br, 4H). 4.30 (br, 4H), 3.69 (br, 4H), 2.24 (br, 4H).

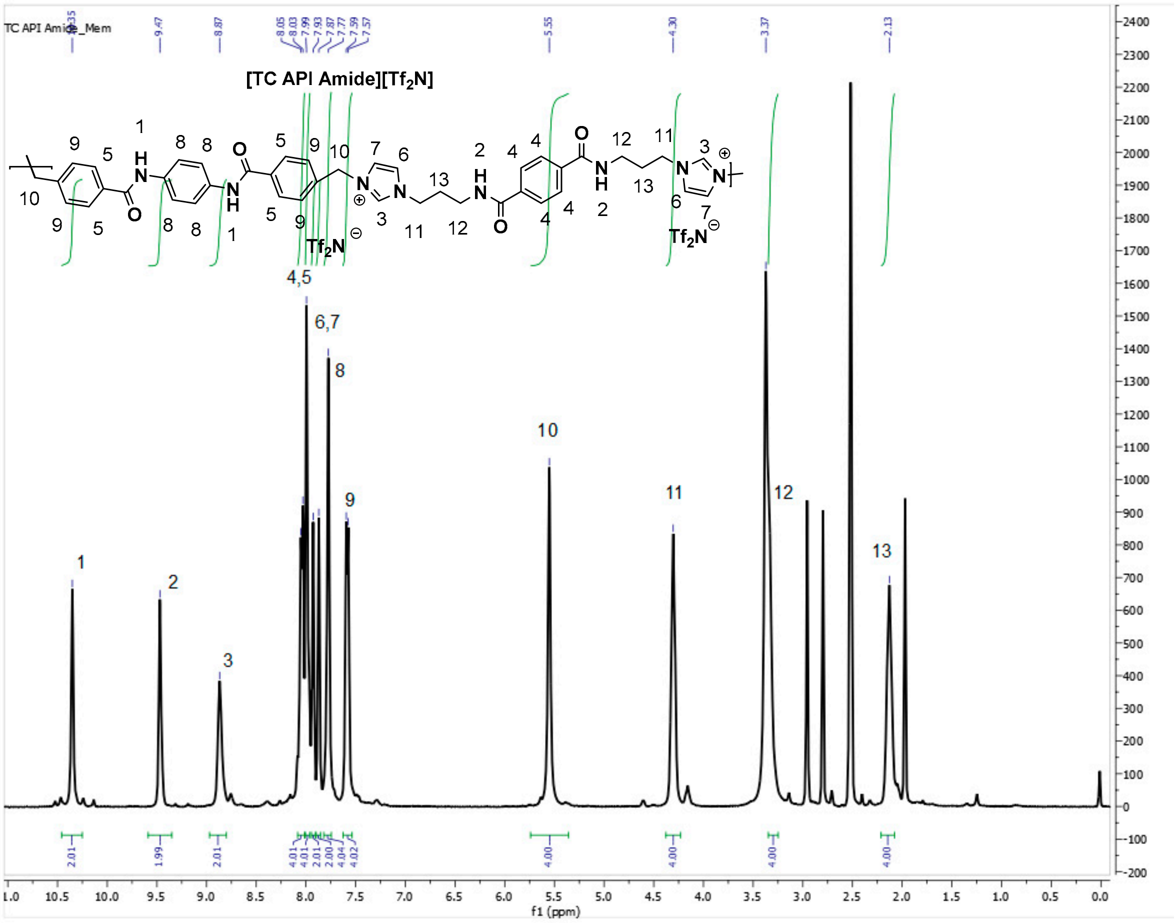


**Figure S2.** $^{1}$H-NMR spectra for [TC API Amide][$Tf_2N$].

$^{1}$ H NMR (500 MHz, DMSO-d6) δ 10.35 (s, 2H), 9.47 (s, 2H), 8.87 (s, 2H), 8.04 (d, J = 7.19 Hz, 4H), 7.99 (s, 4H), 7.93 (s, 2H), 7.87 (s, 2H), 7.77 (s, 4H), 7.58 (d, J = 7.24 Hz, 4H), 5.55 (s, 4H). 4.30 (br, 4H), 3.37 (br, 4H), 2.13 (br, 4H).

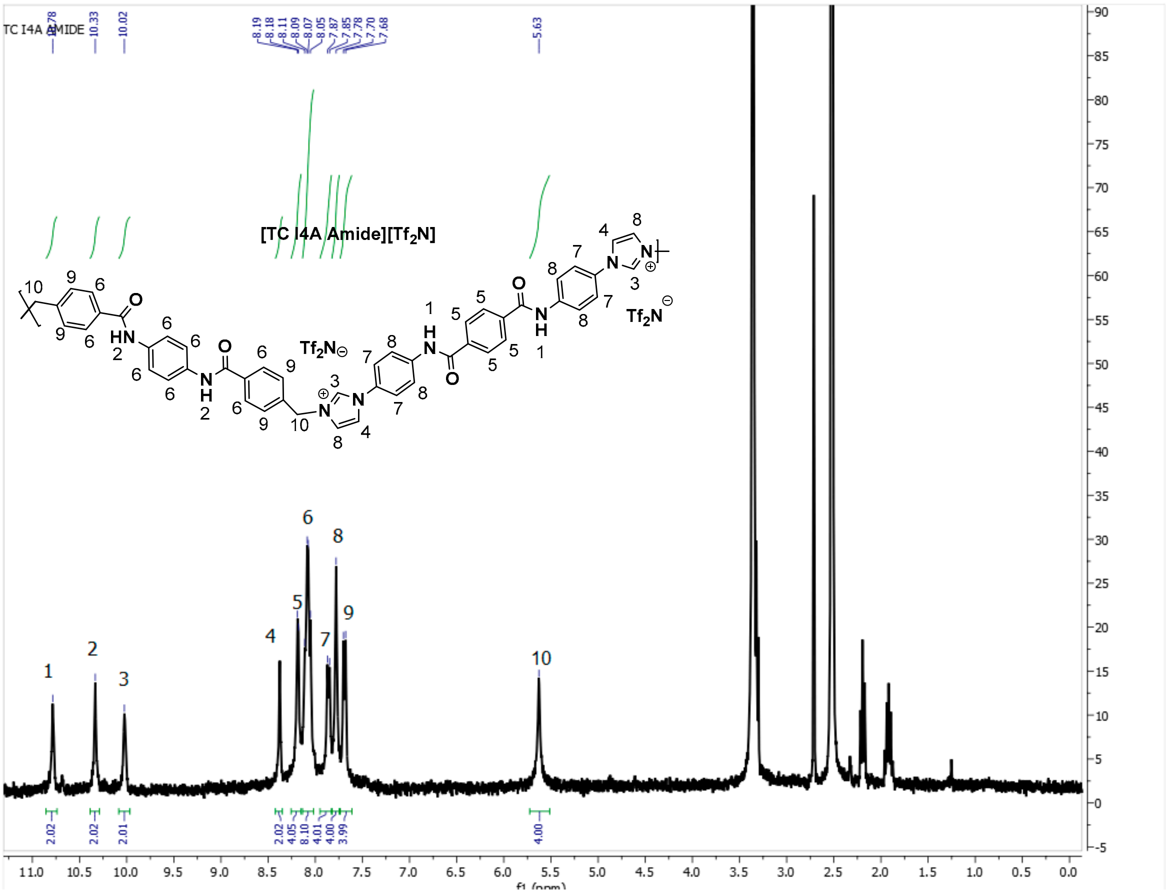

**Figure S3.** $^1$H-NMR spectra for [TC I4A Amide][$Tf_2N$].

$^1$H NMR (500 MHz, DMSO-d6) δ 10.78 (s, 2H), 10.33 (br, 2H), 10.08 (br, 2H), 8.38 (br, 2H), 8.19 (d, J = 3.94 Hz, 4H), 8.08 (q, J = 4.74, 9.11 Hz, 8H), 7.86 (d, J = 8.27 Hz, 4H), 7.78 (br, 6H), 7.69 (d, J = 8.68 Hz, 4H), 5.63 (br, 4H).

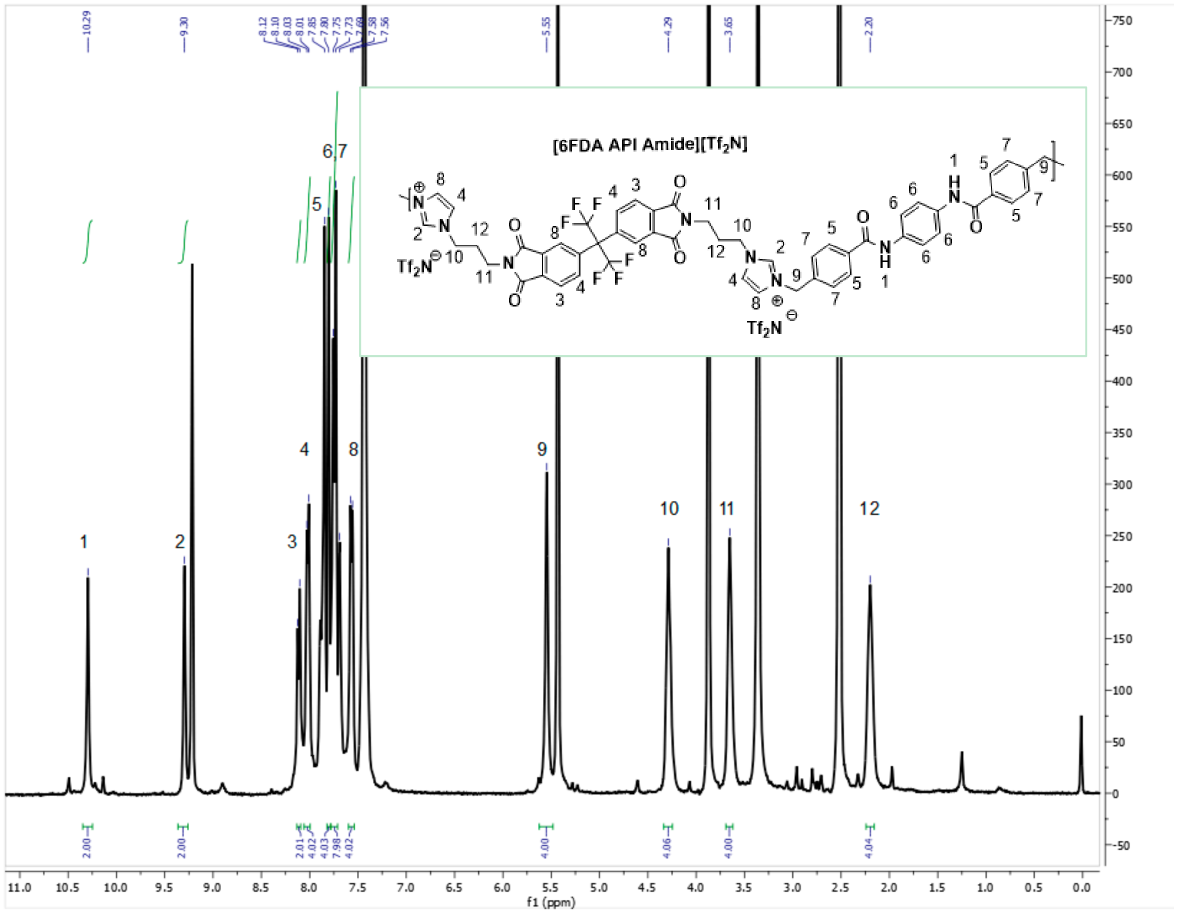

**Figure S4.** $^{1}$H-NMR spectra for [6FDA API Amide][$Tf_2N$].

*Note: Peaks were less broadened in the NMR of the composite than the neat ionene, thus coupling constants were clearer to extract from the above spectrum due to resolution. Thus, [Bnmim][$Tf_2N$] peaks can also be seen on this spectrum.

$^{1}$H NMR (500 MHz, DMSO-d6) δ 10.29 (s, 2H), 9.30 (s, 2H) 8.11 (d, J = 7.79 Hz, 2H), 8.01 (d, J = 7.02 Hz, 2H), 7.95 (m, 6H), 7.90 (br, 2H), 7.80 (s, 4H) 7.73 (s, 4H), 7.75 (s, 4H), 7.57 (d, J = 7.34 Hz, 4H), 5.55 (s, 4H). 4.29 (br, 4H), 3.65 (br, 4H), 2.20 (br, 4H).

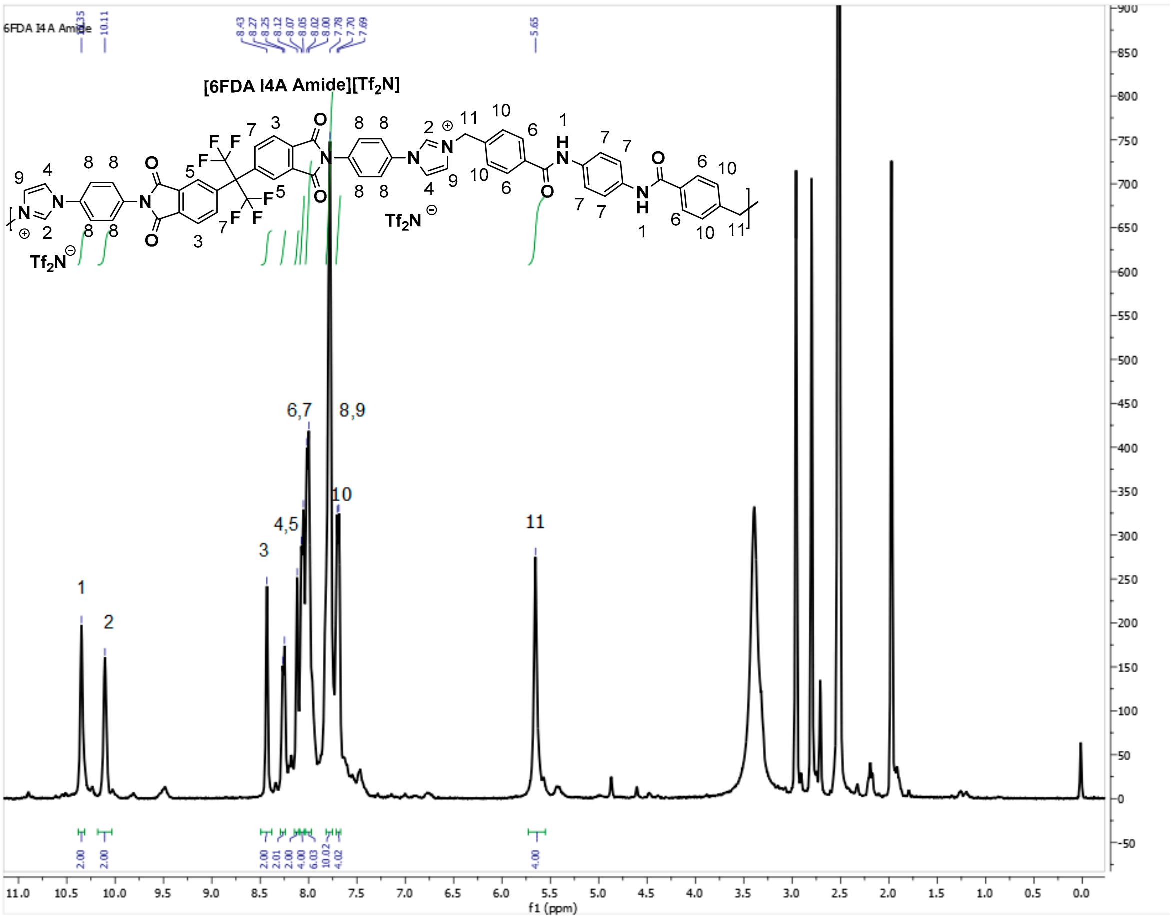


**Figure S5.** $^{1}$H-NMR spectra for [6FDA I4A Amide][Tf2N].

$^{1}$H NMR (500 MHz, DMSO-d6) δ 10.35 (s, 2H), 10.11 (s, 2H), 8.43 (s, 2H), 8.26 (d, J = 6.98 Hz, 2H), 8.12 (s, 2H), 8.06 (d, J = 6.79 Hz, 4H), 8.01 (d, J = 6.90 Hz, 6H), 7.79 (br, 2H), 7.78 (br, 8H), 7.70 (d, J = 6.86 Hz, 4H), 5.65 (s, 4H).

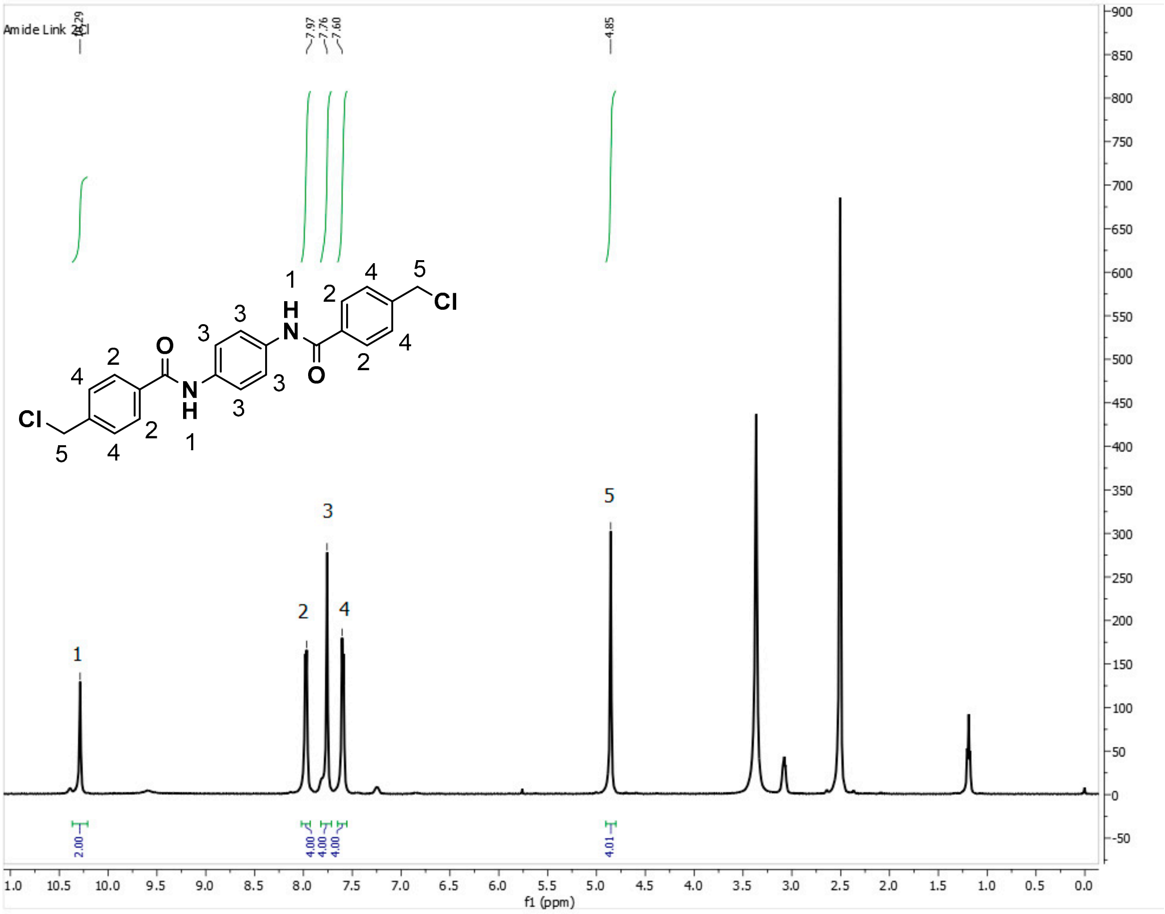


**Figure S6.** $^{1}$H-NMR spectra for diamide dichloride linkage.

$^{1}$H NMR (500 MHz, DMSO-$d_6$) δ 10.29 (s, 2H), 7.97 (d, J = 7.0 Hz, 4H), 7.76 (s, 4H), 7.60 (d, J = 7.27 Hz, 4H), 4.85 (s, 4H).

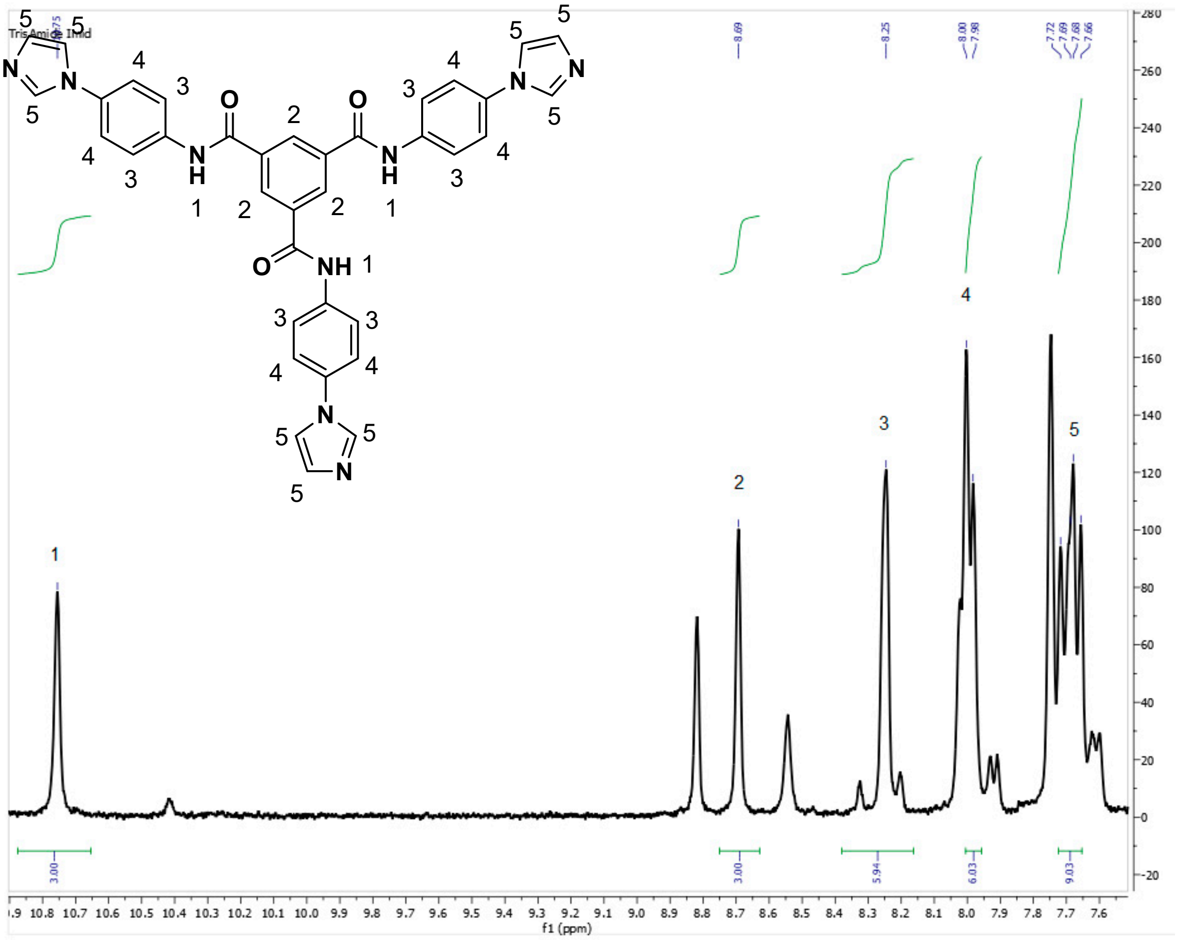

**Figure S7.** $^1$H-NMR spectra for N1,N3,N5-tris(4-(1H-imidazol-1-yl)phenyl)benzene-1,3,5-tricarboxamide. Residual "I4A" is observed, but was removed during the work up of the ionic form.

$^1$H NMR (500 MHz, DMSO-$d_6$) δ 10.75 (s, 3H), 8.69 (s, 3H), 8.25 (s, 3H), 7.99 (d, J = 7.39Hz, 6H), 7.68 (m, J = 8.24, 11.33 Hz, 9H).

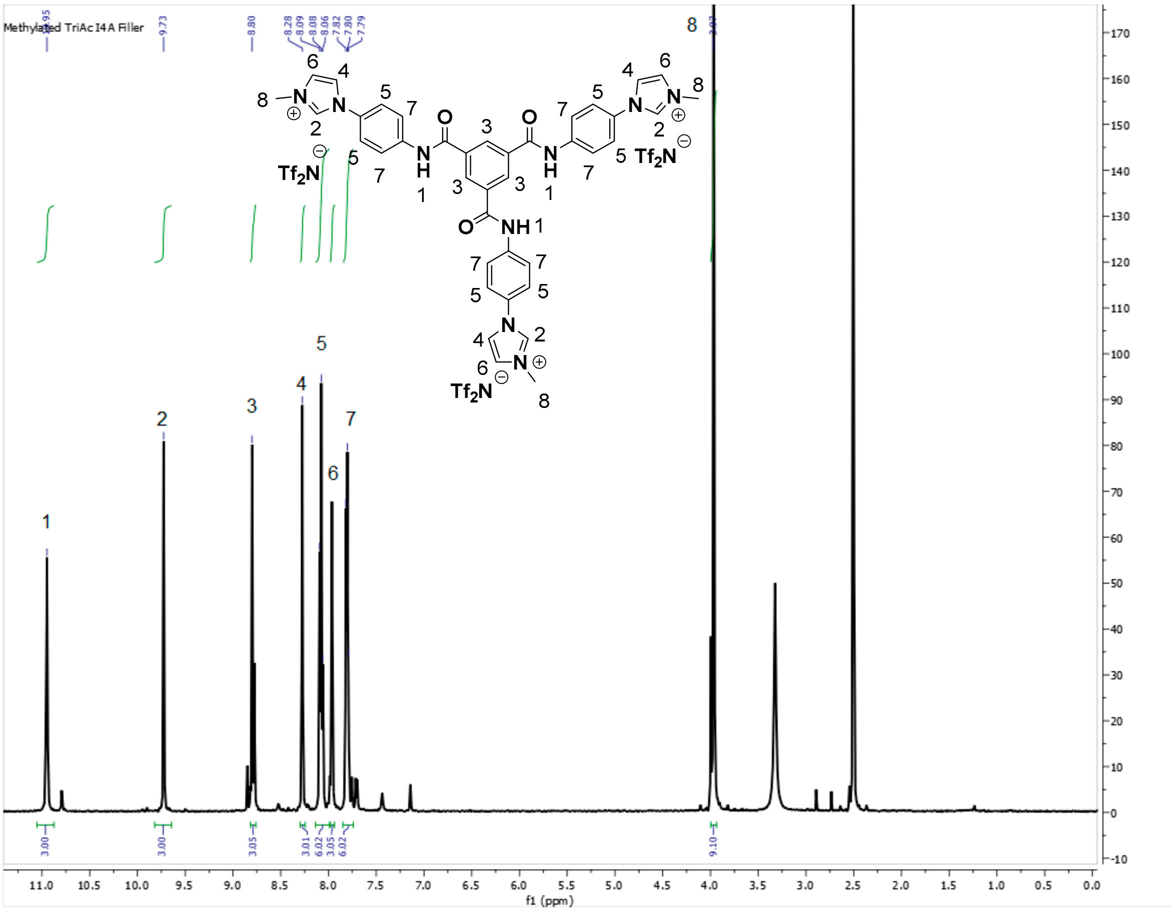


**Figure S8.** $^{1}$H-NMR spectra for 1,1',1''-(((benzene-1,3,5-tricarbonyl)tris(azanediyl))tris(benzene-4,1-diyl))tris(3-methyl-1H-imidazol-3-ium) bistriflimide.

$^{1}$H NMR (500 MHz, DMSO-$d_6$) δ 10.95 (br, 3H), 9.73 (s, 3H), 8.80 (d, J= 10.95 Hz, 3H), 8.28 (m, J = 1.52 Hz, 3H), 8.08 (td, J = 1.21, 8.85 Hz, 6H), 7.96 (m, 3H), 7.81 (td, J = 1.20, 8.76, 6H) 3.97 (s, 9H).

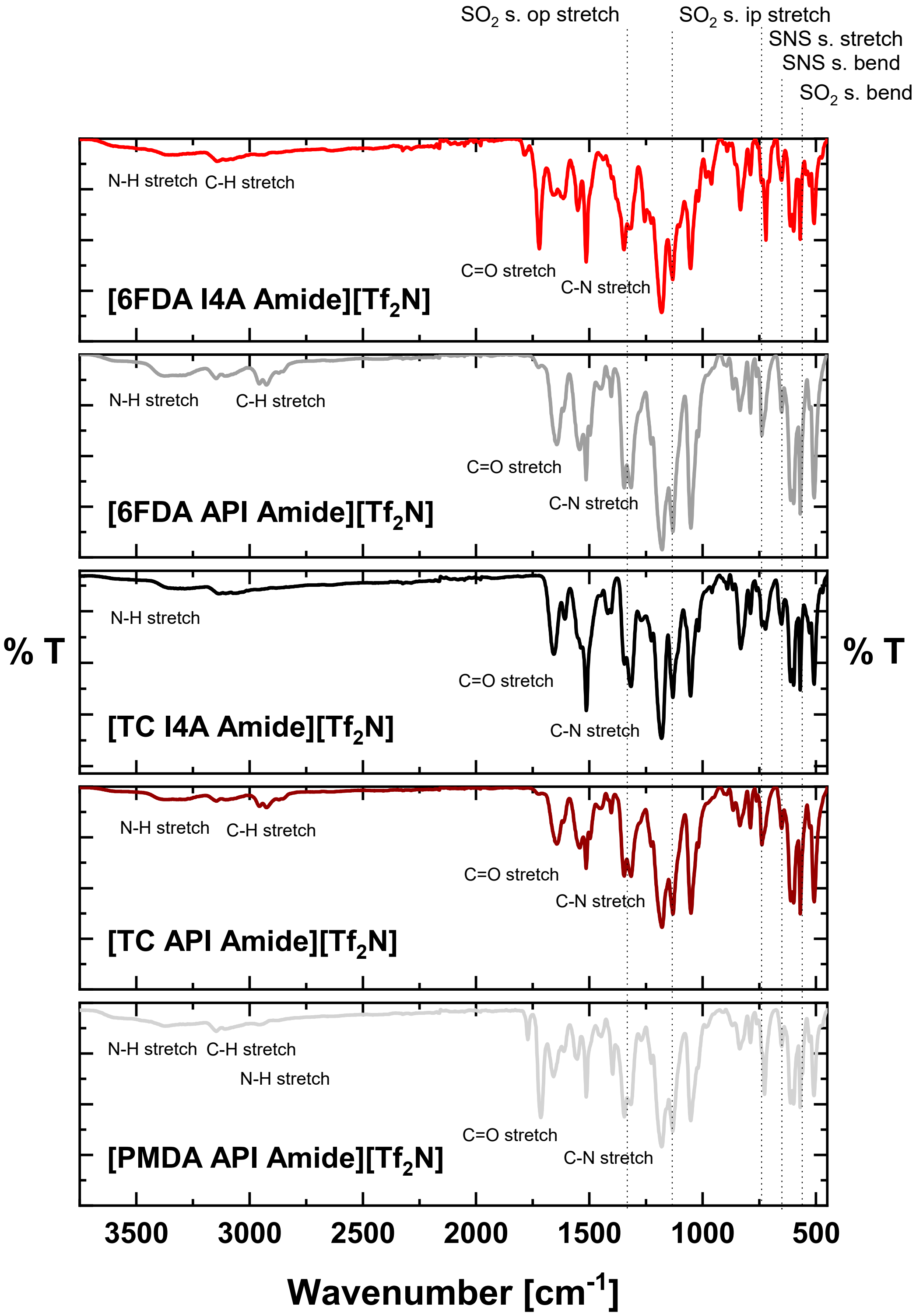


**Figure S9.** FTIR data for five PAA and PAI ionenes, labeled with relevant stretching and bending vibrations. Key functional features of the backbone and the associated anion are highlighted.

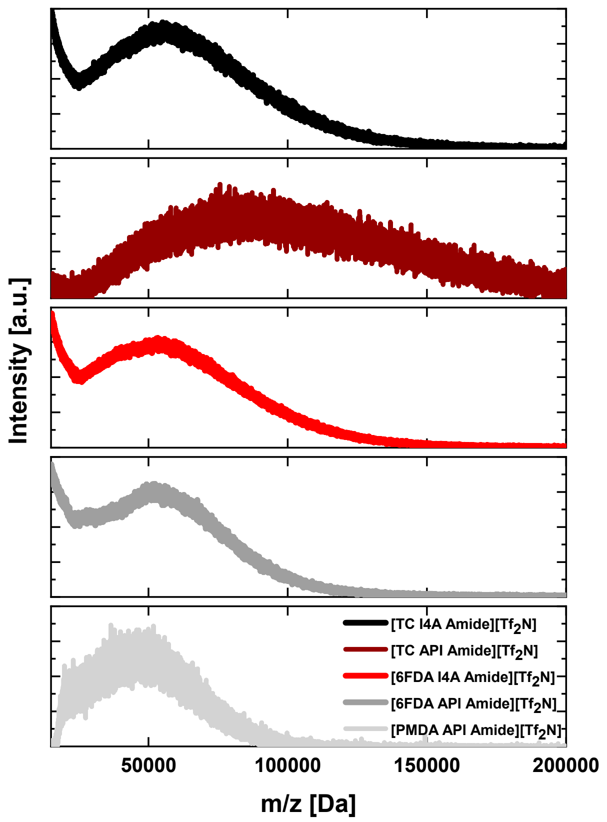


**Figure S10:** MALDI-TOF data for the set of neat ionenes.

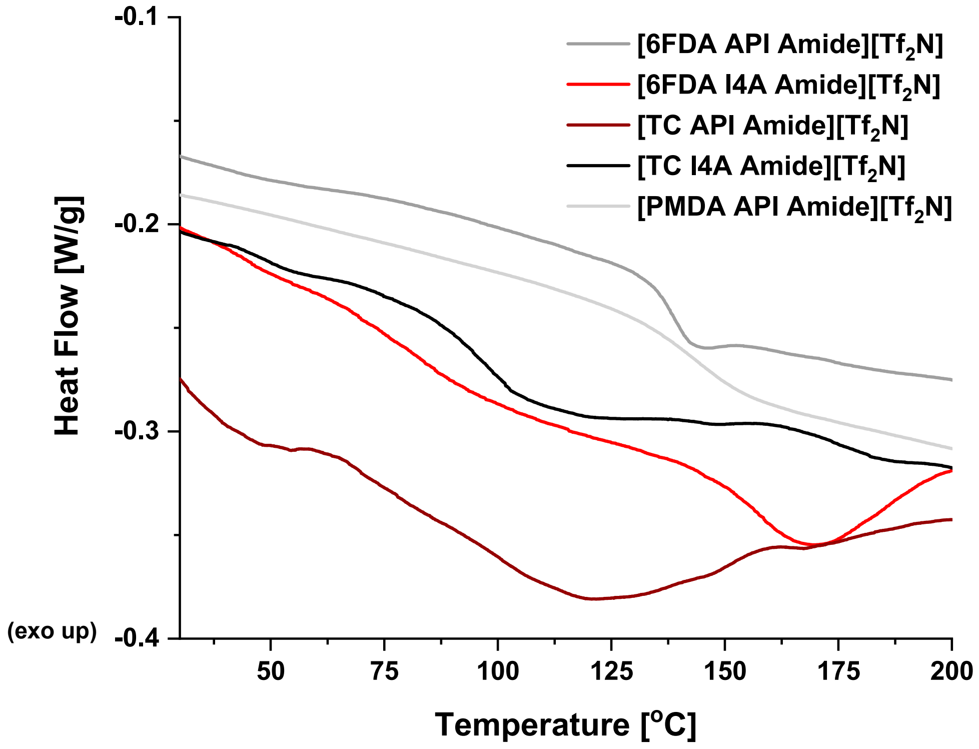


**Figure S11.** DSC plot for five poly(amide-imide) and poly(amide-amide) derivatives showing the range of $T_g$.

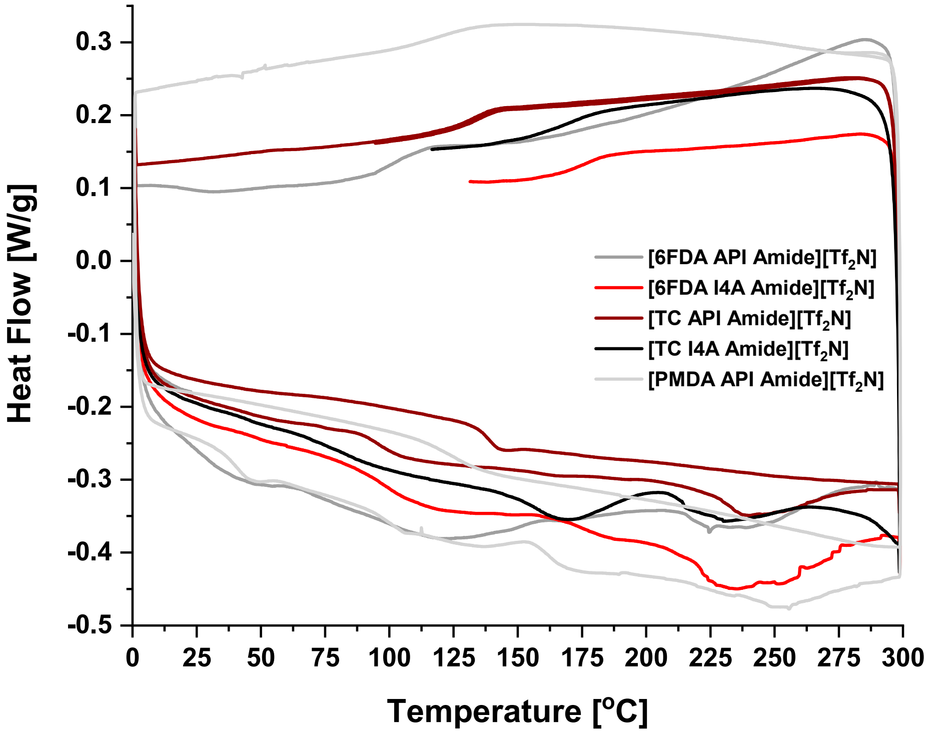


**Figure S12.** DSC plot for five poly(amide-imide) and poly(amide-amide) derivatives showing the full cycles and the range for $T_m$.

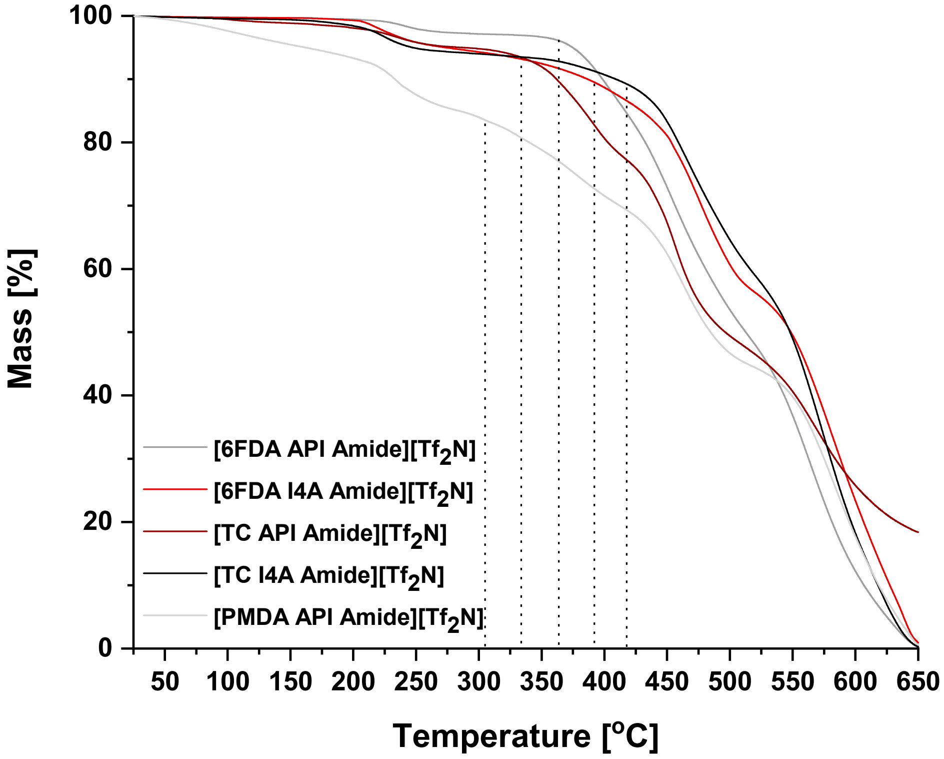


**Figure S13.** TGA data for five poly(amide-imide) and poly(amide-amide) derivatives.

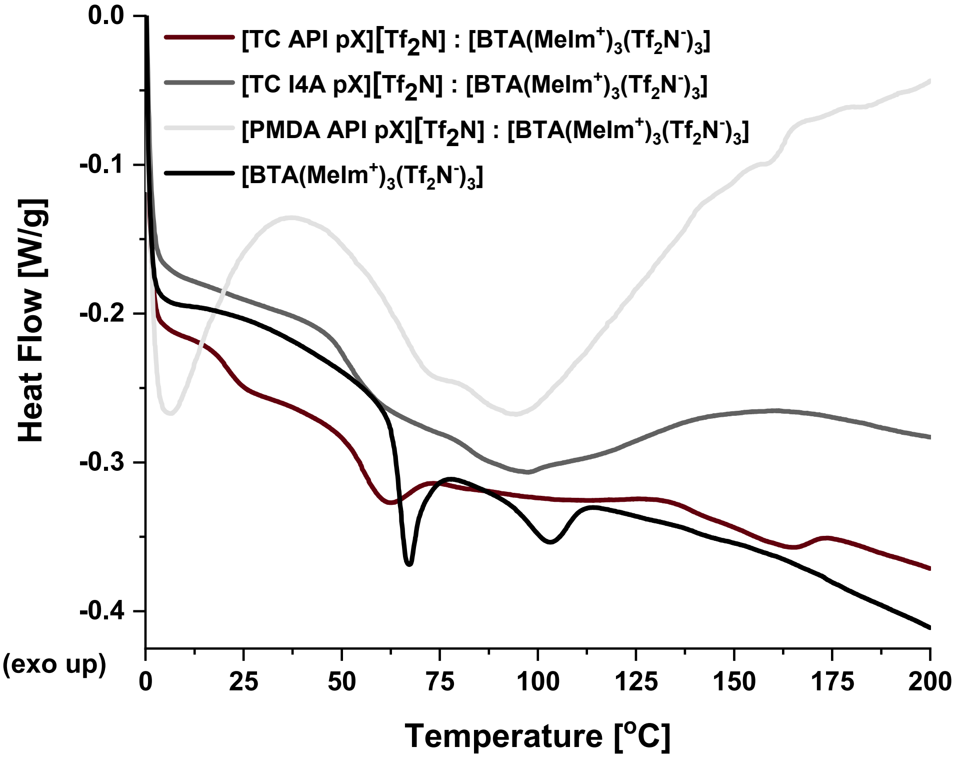


**Figure S14.** DSC data for polyimide and polyamide ionenes containing [BTA(MeIm$^+$)$_3$][Tf$_2$N$^-$]$_3$ .

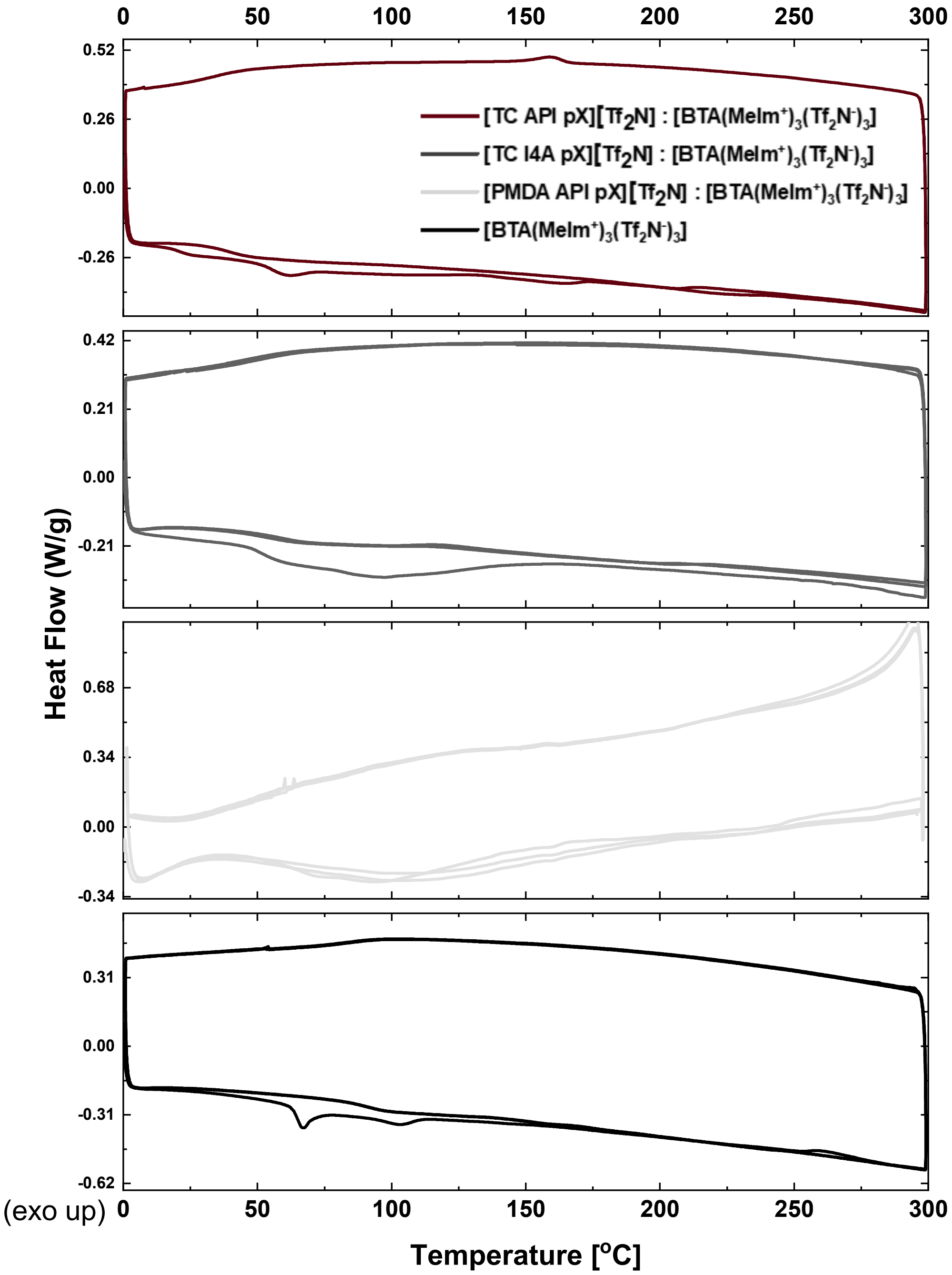


**Figure S15.** DSC full cycles for polyimide and polyamide ionenes containing [BTA(MeIm$^+$)$_3$][Tf$_2$N$^-$]$_3$.

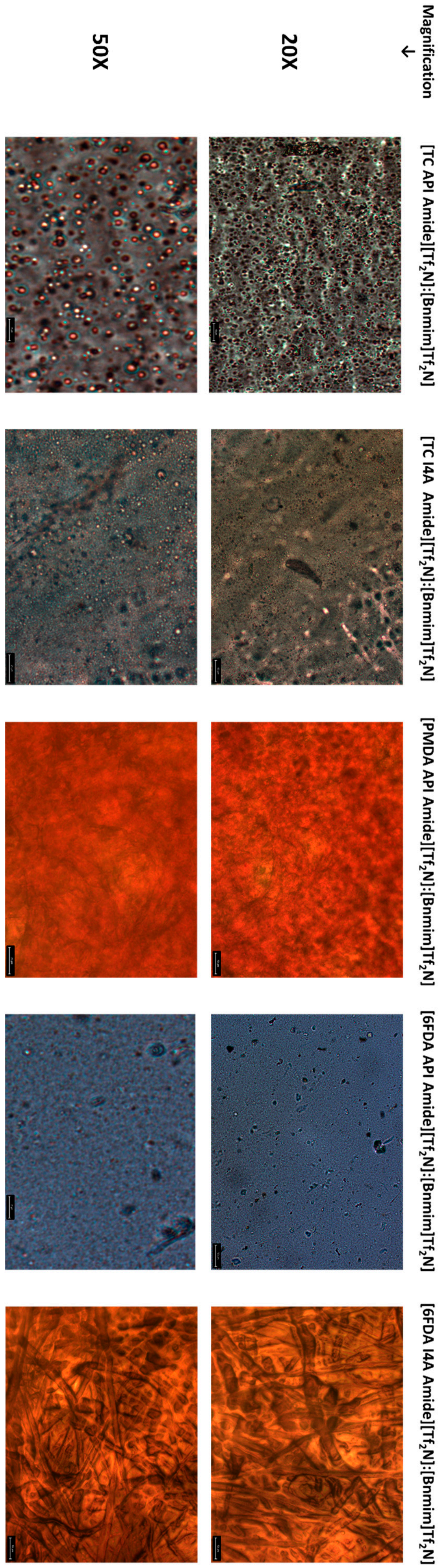


**Figure S16.** PLM images for PAA and PAI ionenes containing [Bnmim][$Tf_2N^-$].
